# Development and validation of a new ICD-10-based screening colonoscopy overuse measure in a large integrated healthcare system: a retrospective observational study

**DOI:** 10.1136/bmjqs-2021-014236

**Published:** 2022-10-03

**Authors:** Megan A Adams

**Affiliations:** VA Ann Arbor Center for Clinical Management Research, VA Ann Arbor Healthcare System, Ann Arbor, MI 48105, USA

**Keywords:** colonoscopy, colorectal cancer screening, quality measurement

## Abstract

**Background:**

Low-value use of screening colonoscopy is wasteful and potentially harmful to patients. Decreasing low-value colonoscopy prevents procedural complications, saves patient time and reduces patient discomfort, and can improve access by reducing procedural demand. The objective of this study was to develop and validate an electronic measure of screening colonoscopy overuse using ICD-10 codes and then apply this measure to estimate facility-level overuse to target quality improvement initiatives to reduce overuse in a large integrated healthcare system.

**Methods:**

Retrospective national observational study of US Veterans undergoing screening colonoscopy at 119 Veterans Health Administration (VHA) endoscopy facilities in 2017. A measure of screening colonoscopy overuse was specified by an expert workgroup, and electronic approximation of the measure numerator and denominator was performed (“electronic measure”). The electronic measure was then validated via manual record review (n=511). Reliability statistics (n=100) were calculated along with diagnostic test characteristics of the electronic measure. The measure was then applied to estimate overall rates of overuse and facility-level variation in overuse among all eligible patients.

**Results:**

The electronic measure had high specificity (99%) and moderate sensitivity (46%). Adjusted PPV and NPV were 33% and 95%, respectively. Inter-rater reliability testing revealed near perfect agreement between raters (k=0.81). 269,572 colonoscopies were performed in VHA in 2017 (88,143 classified as screening procedures). Applying the measure to these 88,143 screening colonoscopies, 24.5% were identified as potential overuse. Median facility-level overuse was 22.5%, with substantial variability across facilities (IQR 19.1%–27.0%).

**Conclusions:**

An ICD-10-based electronic measure of screening colonoscopy overuse has high specificity and improved sensitivity compared to a previous ICD-9-based measure. Despite increased focus on reducing low-value care and improving access, a quarter of VHA screening colonoscopies in 2017 were identified as potential low-value procedures, with substantial facility-level variability.

## INTRODUCTION

Low-value use of colonoscopy for screening and other preventive indications is a common and well-documented problem across healthcare systems.^1–3^ A recent systematic review found substantial overuse of diagnostic testing across all healthcare settings and highlighted the need for health systems, providers, and policymakers to develop and implement effective strategies to curb overuse of low-value diagnostic testing.^4^ Low-value care has been characterized as services that provide little to no benefit to patients, have potential to cause harm, incur unnecessary costs to patients, or waste limited healthcare resources.^5–8^ Low-value use of colonoscopy (i.e., overuse) not only is wasteful and potentially harmful to patients—it also can impede access for patients who need care by unnecessarily increasing endoscopy demand in the setting of limited resources. Low-value care is particularly problematic in large integrated healthcare systems such the Veterans Health Administration (VHA), where reduced access may lead to longer-than-acceptable wait times and impair VHA’s ability to meet its central mission to ensure that United States military veterans are able to access the care they need in a timely manner. Indeed, VHA has previously been scrutinized for prolonged wait times for routine medical care, including elective outpatient procedures such as colonoscopy.^9^

In 2014, VHA was investigated for prolonged wait times for routine medical care including elective outpatient colonoscopies at the Phoenix VA and other sites.^9^ Prior to 2014, our team developed an International Classification of Diseases, Ninth Edition (ICD-9)-based electronic measure to identify potential low-value use of screening colonoscopy in VA endoscopy facilities.^3^ However, interim adoption of International Classification of Diseases, Tenth Edition, Clinical Modification (ICD-10-CM; hereinafter, “ICD-10”) rendered the ICD-9-based measure unusable to monitor overuse as the health system sought to improve access to care.

In this study, we sought to develop and validate an ICD-10-based electronic measure of screening colonoscopy overuse. We then applied this measure to estimate facility-level variation in colonoscopy overuse in VHA for purposes of targeting quality improvement initiatives to reduce overuse. We hypothesized that there would be substantial potential overuse of screening colonoscopy in VHA given inherent challenges in reducing use of low-value services and the lack of specific programs or interventions focused on this topic.^10^ We also hypothesized that our ICD-10-based measure would have similar specificity but enhanced sensitivity compared to the previously developed ICD-9-based measure due to the addition of more specific codes for non-screening indications in ICD-10.

## METHODS

This was a retrospective observational study using VHA administrative, clinical, and laboratory data available in the Corporate Data Warehouse (CDW). Because this work was performed under an operations Memorandum of Understanding with the VA Office of Reporting Analytics, Performance, Improvement and Deployment (RAPID), approval by the Ann Arbor VA Institutional Review Board was not required. VHA is the largest integrated healthcare system in the United States. The study population included Veterans undergoing a screening colonoscopy at one of 119 VHA endoscopy facilities in 2017. For patients who had 2 or more colonoscopies within the 2017 study period, we included only the first (index) procedure because our primary focus was on identifying overuse of routine, ambulatory screening colonoscopies. A small proportion of patients undergo multiple colonoscopies in a short period of time, but in most instances those colonoscopies repeated in the short-term (i.e., <1 yr) are appropriate (e.g., due to poor bowel preparation on the initial procedure, inadequate sedation during the initial procedure, or the need for surveillance of a large, piecemeal polypectomy site to ensure no residual polyp tissue remains).

The study proceeded in 3 steps: (1) measure specification and electronic approximation of the measure using ICD-10-era administrative codes; (2) measure validation via manual record review using a national random sample of colonoscopy cases, with oversampling to ensure adequate numbers of screening colonoscopies; and (3) application of the ICD-10-based electronic measure to all eligible patients undergoing colonoscopy to calculate 2017 VHA screening colonoscopy overuse rates, facility-level variation in overuse, and potential explanatory factors.

### Measure Specification and Electronic Measure Construction

Measure specifications were initially defined by an expert workgroup comprising VA experts in colorectal cancer screening and in performance measurement.^3^ Prior to the initial workgroup meeting, members were provided with relevant literature to review, including U.S. Preventive Services Task Force (USPSTF) guidelines for colorectal cancer screening and Veterans Health Administration (VHA) Colorectal Cancer Screening Directives in effect at the time of measure development. While there were interim updates to USPSTF guidelines between 2013 and 2017,^11^ these updates did not impact the measure specifications (a 2021 update to the USPSTF guidelines recommended changing the age of screening initiation from 50 to 45;^12^ however, this was not the standard of care during our study period). Workgroup members were charged with developing a measure that: (1) was based on high-quality evidence (i.e., the evidence summarized in pre-2021 USPSTF guidelines); (2) maximized specificity at the expense of sensitivity; and (3) could be implemented electronically.

The workgroup began by broadly defining overuse as a screening colonoscopy performed at an inappropriately short interval (e.g., screening colonoscopy performed 5 years after a prior negative screening colonoscopy in an average-risk patient) or in a patient for whom the benefit of screening is low (e.g., screening colonoscopy in an 86-year-old). The workgroup was then asked to more clearly specify the data elements that comprised the measure denominator (the eligible population - Table 1, Denominator) and the numerator (those meeting the measure - Table 1, Numerator). For the numerator, discussion focused on the appropriate cutoffs for age, time interval, and life expectancy. The workgroup then classified each item comprising the numerator as “probable” or “possible” overuse based on the strength of the guideline recommendation and the likelihood of misclassification. The workgroup also specified exclusions from the measure denominator, factors indicating that the colonoscopy was not an average-risk screening procedure such as increased risk for colorectal cancer (e.g., screening colonoscopy in a patient with a family history of colorectal cancer) or ineligibility for screening (e.g., prior total abdominal colectomy).

The workgroup met three times over a 6-month period. Rather than using a formal Delphi process, any potential disagreement was resolved through discussion. Ultimately, consensus was achieved in all elements of the measure specification. The final measure defined by the workgroup identified average-risk screening colonoscopies (comprising the eligible population in the denominator – Table 1) that met one or more criteria for probable or possible overuse (comprising the numerator – Table 1).

#### Electronic Approximation of Measure Denominator:

The measure denominator (prior to application of exclusions to eliminate procedures with indications other than average-risk screening) consisted of all index colonoscopies performed in FY2017 in patients who had not had a colonoscopy within the preceding 12 months. To approximate the measure denominator (Table 1), we first identified all colonoscopies performed in FY 2017 for any indication using Current Procedural Terminology (CPT) and Healthcare Common Procedure Coding System (HCPCS) codes (Appendix 1, Table 5). For patients who had more than one colonoscopy performed in FY 2017, only the first (index) procedure was included in the denominator. Likewise, only patients who had no prior colonoscopy performed in the 12 months preceding the index FY2017 colonoscopy were included in the denominator. This is because when a colonoscopy is repeated within a year, there is a high probability that the repeat procedure was done for a reasonable indication such as inadequate bowel preparation on the prior procedure, sedation intolerance leading to an incomplete procedure, or failure to complete the prior procedure due to technical difficulty. The process of updating the measure specifications to ICD-10 coding was aided by use of the 3M^™^ ICD-10 Code Translation Tool, a proprietary software application that assists in the conversion of ICD-9 based applications to ICD-10.^13^

#### Denominator Exclusions (Exclusion of Non-Average-Risk Screening Colonoscopies):

We then excluded procedures that may have been performed for diagnostic or high-risk screening or surveillance indications, using an approach previously developed and validated by Fisher and colleagues.^14^ First, we excluded patients who had an ICD-10 code for specific gastrointestinal symptoms or for colorectal neoplasia within 12 months of the FY17 colonoscopy (Appendix 1, Table 6). To further increase the specificity of the electronic measure, we also excluded individuals with ICD-9 and ICD-10 codes indicating high risk for colorectal cancer or prior total abdominal colectomy at any time in the prior 10 years (from FY07 to FY17) (Appendix 1, Tables 7a and 7b). Specifically, patients were excluded if CPT or ICD-9/−10 codes revealed any of the following diagnoses between FY07 and the qualifying FY17 colonoscopy (Appendix 1, Tables 7a and 7b): (1) prior colectomy, (2) history of colorectal cancer; (3) history of colon polyps; (4) history of inflammatory bowel disease; or (5) family history of colorectal cancer. Both ICD-9 and ICD-10 codes were used because VHA (like most US healthcare systems) transitioned between these two coding systems on October 1, 2015. These additional exclusion criteria were selected to ensure that the cohort comprised individuals who were at average (rather than increased) risk of CRC. Finally, we excluded individuals who underwent their FY17 colonoscopy during a hospitalization (since such colonoscopies are unlikely to be performed for screening). Thus, the final denominator (after all exclusions) consisted of all average-risk screening colonoscopies performed in FY2017.

#### Electronic Approximation of Measure Numerator:

Specification of electronic elements comprising the measure numerator (probable and possible overuse – Table 1) was more straightforward than for the denominator since these elements were primarily based on factors such as patient age and the time interval between colonoscopies (which are reliably-coded in administrative data). To identify fecal occult blood tests (FOBTs), we used Logical Observation Identifiers Names and Codes (LOINC) (Appendix 1, Table 5). To identify patients with life expectancy <6 months, we used structured data from CDW that is used to indicate limited life expectancy for clinical purposes (i.e., the CDW Health FactorType domain, which contains information about health factors, severity level, and other indicators of health and includes a forecast of the probable outcome of a disease to flag patients with a life expectancy of <6 months).

### Validation of Updated ICD-10 Measure

#### Validation Sample:

The electronic, ICD-10-based measure was validated against the gold standard of manual record review using a national random sample of colonoscopy cases (i.e., patients who had a CPT or HCPCS code for a colonoscopy of any type performed in a VHA facility in 2017) (Appendix 1, Table 5) stratified by VA and VA community care. For purposes of the validation, we oversampled screening colonoscopies within each stratum using new ICD-10 code Z12.11 (denoting a screening indication) with the goal of achieving 50% screening procedures in our sample, and 50% non-screening procedures in our sample. If we had randomly sampled 500 colonoscopies (i.e., 500 patients who had a colonoscopy procedure code in 2017), approximately 25% of our sample would have been screening procedures (according to 2013 data, only a quarter of all colonoscopies are performed for screening), limiting our ability to conduct a robust validation.^3^ Thus, by oversampling screening colonoscopies using new ICD-10 code Z12.11 within the larger national random sample of colonoscopy cases (i.e., patients who had a CPT or HCPCS code for a colonoscopy of any type performed in a VHA facility in 2017), we were able to ensure that roughly 50% of colonoscopies in our validation sample were screening colonoscopies.

In parallel with the present study, we also validated the measure in VA community care data (i.e., via manual review of non-VA endoscopy reports and other data). VA has recently expanded the ability of VA-enrolled Veterans to receive care in non-VA facilities at VA expense.^15^ Therefore, we initially pulled a sample of 1,000 patients – 500 who had a colonoscopy in a VA facility and 500 who had a colonoscopy through VA community care per administrative data. While the initial sample size for the VA measure validation was 500, 11 of the patients who were identified as having their colonoscopy through VA community care were found to have received their procedure at a VA facility on further review. Therefore, these 11 patients were included in our validation of the measure using VA data. Thus, the final validation sample for this study was 511. Validation of the new ICD-10 measure included review of fewer charts than the validation of the previous ICD-9-based measure (3,000),^3^ because the ICD-10-based measure was an update of the previous measure and the same core data elements used in the original 2012 abstraction were used in the 2017 abstraction, with minor changes in how reviewers were instructed to document several of these data elements.

#### Validation Protocol:

Manual record review was performed by Quality Insights, a professional chart abstraction group that performs large-scale, national chart reviews for the VA performance measurement program (VA External Peer Review Program, or EPRP) on an ongoing basis. EPRP uses quality control processes to maximize the consistency and completeness of data collected from VA records. These processes include: (1) internal quality control (IQC) question and mnemonic level analysis; and (2) inter-rater reliability (IRR) assessment.

We refined a standardized electronic health record (EHR) abstraction algorithm to identify overuse measure elements in manual record review, using a progress similar to that used for the ICD-9-based colorectal cancer screening overuse measure.^3^ A 78-question abstraction algorithm (Appendix 2) was developed in collaboration with Quality Insights and EPRP. We first outlined the data elements that would be needed from manual record review to calculate the measure. We then determined which potential data sources could be accessed to retrieve each of these elements (e.g., endoscopy report, primary care clinic note, laboratory data). We also developed a lexicon of potential findings in each of these data sources (e.g., for colonoscopy indication, findings could include average-risk screening, high-risk screening, surveillance, and diagnostic). This process was iterative and collaborative, conducted through a combination of electronic communication and five conference calls (10/2018–2/2019) between the abstraction leads and study team members with clinical expertise in gastroenterology (SS, MA) to enhance its ease of use and reliability. The a priori data elements, data sources, and potential findings were combined into a standardized algorithm for record reviewers.

Prior to beginning chart abstraction, Quality Insights staff provided education to the abstractors via webinar and PowerPoint presentation. This included introducing each step of the abstraction algorithm and clarifying relevant medical terminology. Abstractors were blinded to the electronic measure determination. During the abstraction process, a log of questions from the abstractors was compiled, and these questions were reviewed and resolved by members of the study team on an ongoing basis (SS, MA). A total of 6 trained abstractors (3 Registered Nurses and 3 with a Registered Health Information Administrator credential; average of 15.6 years medical record review experience), utilized the final algorithm/abstraction instrument (Appendix 2) to review 511 records.

#### Inter-rater Reliability Assessment:

Inter-rater reliability (IRR) testing of the final instrument was performed using Cohen’s kappa and Gwet’s AC as measures of agreement. Since the abstraction of all medical records in the validation sample was impractical, IRR testing was performed on a weighted sample of 100 records drawn from the 511-record validation sample (i.e., two reviewers independently abstracted data from the same 100 records, and then their results were compared).

These 100 records included 25 non-screening, 25 screening/non-overuse cases, and 50 screening/overuse cases, randomly sampled from the full 511-record validation sample. With this sample size, we had >80% power to detect a kappa of 0.61 (moderate agreement) or greater. The two IRR reviewers were Registered Nurses and held the Registered Health Information Administrator credential, with extensive medical record review experience.

#### Calculation of Diagnostic Test Characteristics:

The sensitivity, specificity, positive predictive value (PPV) and negative predictive value (NPV) of the electronic measure were assessed. Because we oversampled for screening procedures in the validation sample using the Z12.11 code (meaning that screening prevalence in the validation sample was higher than in the overall sample by design), direct calculations of PPV and NPV would be inaccurate. We therefore used bootstrapping to calculate the PPV and NPV of the electronic measure in resampled subsets of the 511-record validation sample with the same proportion of screening procedures as in the national/overall sample.

### Application of Measure to Assess VHA Screening Colonoscopy Overuse

Following measure validation, we applied the electronic measure to all eligible patients in 2017 to calculate facility-level overuse, and variation in facility-level overuse of screening colonoscopy to identify facilities with high-levels of overuse as candidates for closer inquiry and potential targets of quality improvement efforts. Rates of overuse were reported as a composite of “possible” and “probable” overuse as specified by the expert workgroup (Table 1).

We also used negative binomial regression to model the proportion of overused screening colonoscopies per facility, with the number of overused screening colonoscopies as the outcome and total number of screening colonoscopies as the offset. Facility-level predictors examined included the following: (1) proportion of screening-eligible patients up to date for screening per current guidelines as assessed by chart review via VA’s ongoing EPRP, (2) median number of weeks between a positive FOBT and colonoscopy (“FOBT wait time”), (3) VA facility complexity score (incorporating factors including patient risk, clinical volume, teaching/research activity, and intensive care unit level, rated on a scale from 1a (highest complexity) to 3 (lowest complexity)), (4) academic affiliation (obtained from the VA Office of Academic Affiliations), (5) annual colonoscopy volume (2017, extracted from CDW), and (6) proportion of colonoscopies outsourced to non-VA facilities (2017, extracted from CDW); (7) proportion of colonoscopies performed on Black patients (extracted from CDW), and (8) proportion of colonoscopies performed on Hispanic or Latino patients (extracted from CDW). Risk ratios (RR) and 95% confidence intervals (CI) were calculated. All data management and analyses were performed using SAS, Version 9.4, of the SAS Enterprise Guide for LINUX (SAS Institute Inc., Cary, NC).

## RESULTS

### Validation of Electronic Measure:

Based on independent duplicate manual review (n=100), the kappa of the final instrument was 0.81 (95% CI 0.69–0.93) and the Gwet’s AC was 0.83 (95% CI 0.72–0.94), indicating “near perfect” and “very good” agreement, respectively. Both reviewers identified 35 of 100 cases as screening colonoscopy overuse, and 56 of 100 cases as appropriate colonoscopies. Reviewers disagreed in 9 of 100 cases. In 7 of 9 cases of disagreement, reviewers disagreed regarding whether the procedure was a screening procedure, rather than on whether that screening procedure was overuse. In the other 2 cases of disagreement, the reviewers agreed that the procedure was a screening colonoscopy but disagreed regarding whether that screening colonoscopy was appropriate vs. overuse.

Compared to manual record review (n=511), the ICD-10-based electronic measure had high specificity (99%, 95% CI 98%–100%) and improved sensitivity (46%, 95% CI 35%–57%) compared to the prior ICD-9 based measure (which had a sensitivity of 20% and a specificity of 97%).^3^ The electronic ICD-10-based measure was also accurate in estimating overuse compared to manual record review (19% ICD-10-based measure overuse (95% CI 15–24%) vs. 23% manual record review overuse (95% CI 19–28%)). The adjusted PPV of the electronic measure was 33% (95% CI 21%–42%) and the adjusted NPV was 95% (95% CI 93%–97%).

### Measurement of Overuse in VHA:

A total of 269,572 outpatient colonoscopies were performed in VHA in 2017 (36% screening, 64% non-screening indications). After applying exclusion criteria, 88,143 screening colonoscopy encounters remained. Patients were predominantly male (91.6%) and healthy (median Charlson-Deyo comorbidity index score^16^ = 0), with a median age of 62 (interquartile range (IQR) 54–68). (Table 2) Facilities were predominantly academically affiliated (95.0%) and high-complexity (64.7%).

**Figure 1.**
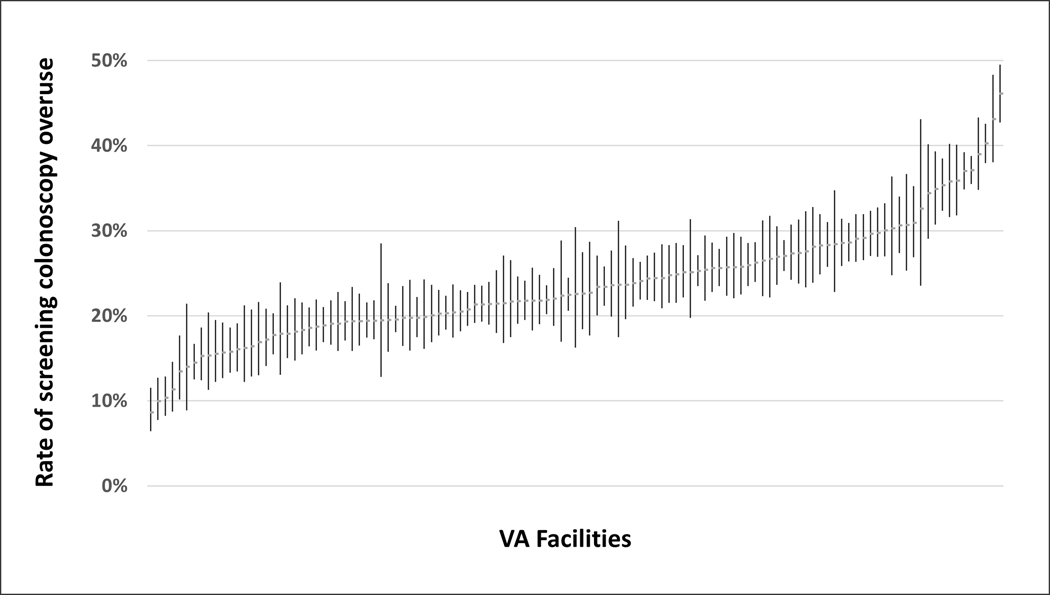
Variation in overuse of screening colonoscopy across 119 Veterans Health Administration facilities (N=88,143). Each marker represents a single VA facility, with error bars indicating 95% confidence intervals (median overuse = 23%, interquartile range = 19% to 27%). (Created by the authors)

Applying the electronic measure to all eligible patients in 2017, 24.5% (21,600/88,143) of VA screening colonoscopy encounters in 2017 met the definition for probable (13.3%, 11,759) or possible (11.2%, 9,841) overuse. Of the 21,600 colonoscopies meeting a consensus definition of overuse, the top two reasons for overuse were screening colonoscopy performed <9 years after a previous colonoscopy (45% in 2017) and screening colonoscopy performed <6 months after a negative FOBT (23% in 2017). (Table 3) Median facility-level overuse was 22.5% (IQR 19.1%–27.0%), with four- to five-fold variability among facilities based on crude percentages. (Figure 1)

### Examining Predictors of Overuse:

Examining the association between screening colonoscopy overuse and facility characteristics, none of the facility-level factors examined were found to be associated with screening colonoscopy overuse except academic affiliation (RR 1.41, 95% CI 1.06–1.87). (Table 4)

## DISCUSSION

In this study, we developed and validated a new ICD-10 based measure of screening colonoscopy overuse and demonstrated that it measures overuse with robust specificity and markedly better sensitivity than a previous ICD-9-based measure.^3^ The new ICD-10-based measure could be used to track facility-level screening colonoscopy overuse over time in the ICD-10 era with little to no burden to clinicians or patients. Such information can be used to limit low-value colonoscopies, thus resulting in both improved quality and expanded capacity for high-value colonoscopies. This is particularly important in systems where access to colonoscopy is limited. Decreasing screening colonoscopy overuse also saves patient time and the anxiety, stress and discomfort associated with undergoing an invasive procedure. Despite increased focus on reducing low value care and enhancing access, approximately 24% of screening colonoscopies in VHA in 2017 were identified as potential low-value procedures with substantial facility-level variability. The ICD-10-based measure was substantially more sensitive in identifying overuse than the previous ICD-9-based measure,^3^ meaning that it detects more potential cases of overuse. While drawing direct comparisons between 2013 and 2017 data has limitations given differences in ICD-9 and ICD-10 measure characteristics, it is worth noting that screening colonoscopy overuse rates did not meaningfully change between 2013 (as measured by an ICD-9 based measure) and 2017 (as measured by an ICD-10 based measure).^3^ This rate of potential overuse is within the credible range found in non-VA health systems in the ICD-9 era in a recent systematic review.^17^

High-rates of potential low-value screening colonoscopy across VHA medical centers (i.e., approximately 1 in 4 screening procedures) may result in part from the absence of assessment of colonoscopy overuse in VHA’s centralized performance measurement and improvement infrastructure. However, in response to high-profile access challenges, there has been increased focus by VHA leadership since 2017 on proposing innovative solutions to address these access challenges, including a focus on reducing procedural overuse. For example, in response to ongoing specialty care backlogs, the VHA Office of Veterans Access to Care (OVAC) convened a VA GI Access Meeting in Washington, DC in September 2018 focused specifically on development and implementation of a coordinated, multi-component access strategy to reduce wait times. This included development and implementation of guidelines and strategies to address overuse, including facility-level monitoring over periods of time (e.g., quarterly) with targeted interventions for sites with relatively high levels of overuse. This work remains ongoing at the national VHA level in conjunction with the VA Office of Reporting, Analytics, Performance, Improvement and Deployment and other operational offices, with continued efforts focused on implementing this ICD-10-based measure into national reporting systems and development of facility-specific reports that can be utilized to communicate performance data to sites and explicitly highlight the link between reducing low-value colonoscopy and improving overall endoscopy access. Thus, there is reason for optimism regarding VHA’s ability to achieve a meaningful reduction in low-value screening colonoscopy in the future. To our knowledge, other well-regarded integrated healthcare systems, including the Kaiser Permanente integrated managed care consortium, do not presently employ a screening colonoscopy overuse monitoring program. Our updated colonoscopy overuse measure could easily be adopted in these non-VHA settings to improve access and enhance overall performance. In addition, non-integrated healthcare systems, such as academic medical centers, community practices, and others could benefit from monitoring of screening colonoscopy overuse, particularly as healthcare reimbursement systems shift to value-based payment/alternative payment models.

The substantial variability in performance across VHA’s 119 endoscopy facilities may be reflective of cultural or other unmeasured facility-level characteristics that enhance or impede efforts to reduce low-value care at those sites. Indeed, substantial variability in use of other low-value diagnostic testing has been demonstrated across facilities in other studies.^18–19^ For example, sites may have varying levels of available resources to carefully triage colonoscopy consults to detect low-value procedures, differences in leadership support and stakeholder engagement to support these efforts, and varying levels of recognition of the strong link between minimizing low-value care (i.e., decreasing demand) and improving overall endoscopy access. Because overuse is a complex problem, accomplishing meaningful and sustainable improvements in facility-level performance will require not only rigorous performance measurement and performance feedback, but also collaboration with willing leaders and frontline providers and patients to facilitate necessary changes to organizational culture.^20^

Our study adds meaningfully to existing knowledge in several ways. First, these findings are among the first to suggest that ICD-10 codes can substantially improve the performance characteristics of electronic quality measures.^21^ The markedly improved sensitivity of our updated colonoscopy overuse measure was largely due to significant improvement in the sensitivity of the underlying electronic measure for screening indication due to the addition of more specific codes for non-screening indications in ICD-10. Specifically, the sensitivity of the ICD-9-based electronic measure for screening indication was only 36%,^3^ as compared to 79% for the ICD-10-based measure. Examples of cases that might still be missed by the ICD-10-based measure include situations in which an overuse screening colonoscopy is misclassified as non-screening (e.g., due to a diagnostic code inappropriately leading to exclusion—see Appendix 1) or in which a patient undergoes colonoscopy outside the VA healthcare system (which is not captured electronically in VA data). Second, development and validation of an ICD-10-based electronic measure will allow health systems (whether in VHA, other integrated health care systems, academic centers, or community practices) to monitor overuse rates longitudinally in the ICD-10 era, which is essential for tracking performance over time and targeting interventions to low performing sites. The majority of performance measures track underuse rather than overuse,^22^ making this measure one of only a handful of overuse measures that can be implemented in clinical performance monitoring programs to enhance healthcare value and optimize access.

Applying such a measure at the health system level can be particularly effective, because health systems can leverage efficiencies of scale to apply such metrics across a large number of affiliated clinicians, use such measures to track performance over time, and develop and disseminate new programs or technologies (including clinical decision support tools, evidence-based guidelines, or other tools) to improve system-wide care delivery and change organizational culture to value stewardship of healthcare resources.^23^ As an electronic measure, the screening colonoscopy overuse measure could be easily integrated into electronic-health record (EHR) platforms such as Epic or Cerner, and used in clinical decision support (i.e., at the time a procedure is ordered) to flag a potential low-value procedure or used for quality monitoring and improvement efforts such as what is being considered in VHA. While this measure is currently not included in national performance programs such as the Merit-Based Incentive Payment System (MIPS) or other pay-for-performance programs, which have tended to favor more typical underuse measures, widespread adoption of a screening colonoscopy overuse or other low-value care measure in this manner could aid in dissemination across practice settings and enhance its potential impact.

We acknowledge that varying incentives (including financial incentives) may influence the degree of motivation present in these various practice settings to adopt such low-value care measures into routine performance monitoring programs. However, adoption of alternative payment models that modify these organizational incentives could be effective at reducing low-value care, as suggested by a recent study of variation in use of low-value services in provider organizations.^24^

Several study limitations also are worthy of mention. First, our measure relies on accurate coding of colonoscopy indication in administrative data, which is difficult to verify. However, comparing our measure to medical record abstraction did show high levels of accuracy. Further, facility-level variation in screening colonoscopy overuse rates may have been confounded by unmeasured factors including the potential for systematic differences in coding of procedural indication across sites and existing implementation of facility-specific interventions designed to curb screening colonoscopy overuse. The potential for coding differences across sites is mitigated, however, by use of automated endoscopy reporting software across VA endoscopy sites that auto-populates ICD/CPT codes into the reports. It also is important to acknowledge that our measure is a composite measure that combines cases of “possible” and “probable” overuse. However, prioritization of measure elements is possible (e.g., prioritizing efforts towards facilities with the highest rates of probable overuse) and is being discussed as part of our efforts to use this measure for quality improvement within the VHA. Additionally, some reasons for overuse (e.g., repeat colonoscopy 5 years after prior negative colonoscopy) are arguably more important to target than others. Finally, we acknowledge that there can never be perfect adherence to guidelines, both because guidelines themselves are not perfect and because guidelines must be applied in the context of the individual patient. While the “acceptable” level of overuse has not been precisely defined, this measure can aid in identifying outlier facilities whose practices should be more closely scrutinized.

With respect to our analysis of facility-level predictors of screening colonoscopy overuse, we acknowledge that, because some of the confidence intervals are wide and the point estimates substantial, there could be associations between overuse rates and facility-level predictors other than academic-affiliation that were not detected in our analysis. However, it is not possible to increase the power of our study because the number of VHA endoscopy facilities is fixed.

Therefore, while this component of our analysis may leave some unanswered questions, we believe the findings still add substantially to the literature and presenting our results is valuable to the field. Certainly, as the largest integrated healthcare delivery system in the United States, VHA is an optimal setting in which to conduct this type of analysis. It is also important to recognize that, while our study demonstrates variation in facility-level overuse, the drivers of overuse among individual low-performing facilities may be quite different, such that tailored strategies to reduce low-value use of screening colonoscopy will likely be needed rather than a uniform approach. We also could have underestimated the true rate of screening colonoscopy overuse among VA-enrolled Veterans by not capturing use of non-VA care, which is increasingly prevalent given legislative initiatives such as the VA Maintaining Internal Systems and Strengthening Integrated Outside Networks (MISSION) Act of 2018^15^ and its predecessors. However, we were able to capture these cases in our manual record review. We also separately validated our measure using VHA community care data, suggesting the measure could be applied to data and sites outside of VHA. Finally, emerging changes in screening colonoscopy guidelines, including a newly updated USPSTF recommendation (released May 2021) to begin colorectal cancer screening at age 45 (rather than 50) in average risk patients, may require refinement of overuse criteria in the future to ensure that the measure does not penalize appropriate care.^12^ However, it is important to note that only a small proportion (12%) of potential overuse was due to performance of a screening colonoscopy in patients 45–49 years of age.

## CONCLUSION

Our updated ICD-10 based measure reliably measures screening colonoscopy overuse with similar specificity but markedly better sensitivity than a previous ICD-9-based measure, allowing VHA to track facility-level performance over time and target sites with higher rates of low-value procedures for improvement. Despite increased focus on reducing low value care and enhancing access, levels of screening colonoscopy overuse in VHA were substantial in 2017, with significant facility-level variability. None of the facility-level factors examined were found to be associated with screening colonoscopy overuse, except for academic affiliation. However, recent systematic efforts to address specialty care access barriers, including through reducing procedural overuse, reflect increasing recognition of the impacts of overuse on overall procedural access and hold promise for reducing overuse of procedural services such as low-value screening colonoscopies in the future.

## Supplementary Material

Supp1 Appendix 1. Additional details regarding electronic approximation of measure denominator and measure numerator.

Supp2 Appendix 2. Abstraction instrument for manual record review.

## Figures and Tables

**Table 1.** Numerator, Denominator and Exclusions for Screening Colonoscopy Overuse Measure

**Numerator:**	**“Probable” overuse of screening colonoscopy:**
	(1) Colonoscopy performed less than 9 years after complete colonoscopy (N=9,692). (2) Colonoscopy performed in a patient < 40 or > 85 years of age (N=1,959). (3) Colonoscopy performed in a patient with life expectancy < 6 months (N= 108).^#^ (4) Colonoscopy performed < 6 months after negative fecal occult blood test (FOBT) (N=4,906).
	**“Possible” overuse of screening colonoscopy:**
	(1) Colonoscopy performed in a patient 40 to 49 years of age (N=3,470). (2) Colonoscopy performed in a patient 76 to 85 years of age (N=1,465).
**Denominator:**	Patients who underwent an index colonoscopy in FY2017 and did not have a colonoscopy in the preceding 12 months. All colonoscopies performed in all patients in FY2017 (N=269,572) First (index) colonoscopy in FY2017 for patients with >1 (N=255,541) Index colonoscopies in patients without colonoscopy in prior 12 months (N=247,155) [Denominator after exclusions below applied (i.e., average-risk screening colonoscopies): N=88,143]
**Exclusions:**	
	Colonoscopy performed for an indication other than average-risk screening (N=159,012). (1) Colonoscopy performed for a diagnostic, high-risk screening, or surveillance indication (N=72,657). * (2) Colonoscopy performed in a patient at increased risk for colorectal cancer (N=85,242).^+^ (a) Personal history of adenomatous polyps (N=77,962). (b) Personal history of colorectal cancer (N=1,488). (c) Personal history of inflammatory bowel disease (N=310). (d) Family history of colorectal cancer (N=5,482). (3) Colonoscopy performed in a patient who has undergone prior total abdominal colectomy (N=1)^+^ (4) Colonoscopy performed during hospitalization (N=1,112)

# Using the HealthFactorType domain in the VA Corporate Data Warehouse (CDW), which contains information about health factors, severity level, and other indicators of health, and includes a forecast of the probable outcome of a disease to flag patients with an expected life expectancy of <6 months.

* Using ICD-9, and ICD-10 codes from FY16 to FY17 (Appendix 1, Table 6).

+ Using CPT, ICD-9, and ICD-10 codes from FY07 to FY17 (Appendix 1, Tables 7a and 7b).

**Table 2.** Characteristics of patients undergoing (N=88,143) and facilities performing (N=119) screening colonoscopies in the Veterans Health Administration, 2017.

Patient Characteristics	
**Age (N (%))**	
<40	1,845 (2.1%)
40–49	3,841 (4.4%)
50–75	80,418 (91.2%)
76–85	1,925 (2.2%)
>85	114 (0.1%)
**Median age (IQR)**	62 (54–68)
**Gender (N (%))**	
Male	80,780 (91.6%)
Female	7,363 (8.4%)
**Charlson comorbidity index score (N (%))**	
0	40,493 (45.9%)
1	20,660 (23.4%)
2	9,484 (10.8%)
3	7,931 (9.0%)
>=4	9,575 (10.9%)
**Race**	
Black	20,106 (22.8%)
Other*	63,736 (72.3%)
Missing	4,301 (4.9%)
**Ethnicity** ^+^	
Hispanic or Latino	5,231 (5.9%)
Not Hispanic or Latino	80,293 (91.1%)
Missing	2,619 (3.0%)
**Facility Characteristics**	
**Academic Affiliation (N (%))**	
Yes	113 (95.0%)
No	6 (5.0%)
**Facility Complexity (N (%))**	
High (Level 1a, b, c)	77 (64.7%)
Medium (Level 2)	24 (20.2%)
Low (Level 3)	18 (15.1%)
**Proportion of colonoscopies performed on Black patients** (median % (IQR))	16 (6–31)
**Proportion of colonoscopies performed on Hispanic or Latino patients** (median % (IQR))	2 (1–6)
# **colonoscopies performed in 2017 for any indication (median (IQR))**	2,052 (1,194–3,045)
**Proportion of individuals up to date with screening (median % (IQR))**	83 (80–86)
**FOBT wait time, in weeks (median (IQR))**	7 (5–9)
**Proportion of colonoscopies “outsourced” to community care (median % (IQR))**	13 (4–29)

* For race, the “Other” category includes “American Indian or Alaska Native,” “Native Hawaiian or Other Pacific Islander,” and “White.”

+ In CDW, ethnicity is reported in only two categories: “Hispanic or Latino” or “Not Hispanic or Latino.”

**Table 3.** Reasons for Screening Colonoscopy Overuse in the Veterans Health Administration, 2017 (N=21,600).

	Definition of Overuse	Number of Colonoscopies (%)
**Probable Overuse**	Less than 9 Years After Negative Colonoscopy	9,692 (44.9)
	Less than 6 Months After Negative FOBT	4,906 (22.7)
	Age < 40	1,845 (8.6)
	Age > 85	114 (0.5)
	Life Expectancy < 6 Months	108 (0.5)
**Possible Overuse**	Age 40–44	905 (4.0)
	Age 45–49	2,565 (12.0)
	Age 76–85	1,465 (6.8)

**Table 4.** Association between facility-level factors and screening colonoscopy overuse (N=119) in the Veterans Health Administration, 2017.

Effect	Risk Ratio (RR)	95% Confidence Interval (CI)
**Proportion of colonoscopies performed on Black patients (per 1%)** *	1.00	1.00–1.01
**Proportion of colonoscopies performed on Hispanic or Latino patients (per 1%)** *	1.00	1.00–1.01
**Facility Complexity** ^+^		
2 vs. 3	0.90	0.74–1.10
1c vs. 3	0.98	0.79–1.22
1b vs. 3	0.88	0.69–1.13
1a vs. 3	0.89	0.72–1.11
**Academic Affiliation,** yes vs. no	**1.41**	**1.06–1.87**
**Colonoscopy volume (per 100)** ^^^	1.00	1.00–1.01
**Proportion of individuals up to date with screening**	1.00	0.99–1.02
**FOBT wait time (per week)** ^^^	1.00	0.97–1.01
**Proportion of colonoscopies “outsourced”**	1.00	1.00–1.01

* RRs are given for every 1% increase in the proportion of procedures performed on Black patients and Hispanic or Latino patients.

+ The 2011 VA facility complexity model score incorporates factors including patient risk, clinical volume, teaching/research activity, and ICU level, rated on a scale from 1a (highest complexity) to 3 (lowest complexity)).

^ RRs are given for every 100-procedure increase in colonoscopy volume and for every 1 week increase in FOBT wait time.

## What is already known on this topic?

Low-value use of colonoscopy for screening and other preventive indications is a common and well-documented problem across healthcare systems.

## What does this study add?

An ICD-10-based electronic measure demonstrated high specificity and moderate sensitivity for identifying screening colonoscopy overuse in the Veterans Health Administration (VHA), the largest integrated healthcare system in the United States. Levels of screening colonoscopy overuse in VHA were substantial in 2017, with significant facility-level variability.

## How might this study affect research, practice, or policy?

Tracking rates of potential screening colonoscopy overuse over time will allow health systems to target quality improvement interventions to low-performing sites to reduce procedural overuse and enhance overall endoscopy access by eliminating unnecessary colonoscopies.
