# Supplementary material for: Development and validation of a new ICD-10-based screening colonoscopy overuse measure in a large integrated healthcare system: a retrospective observational study
Source: BMJ Qual Saf. Author manuscript; Available in PMC 2023 Jul 10. (PMC10294020; doi:10.1136/bmjqs-2021-014236)
Supplement: Supp1 — Appendix 1. Additional details regarding electronic approximation of measure denominator and measure numerator. [file NIHMS1894124-supplement-Supp1.pdf]

Appendix 1. Additional Details Regarding Electronic Approximation of Measure Denominator and Measure Numerator.

Electronic Approximation of Measure Denominator

The measure denominator (prior to application of exclusions to eliminate procedures with indications other than average-risk screening) consisted of all index colonoscopies performed in FY2017 in patients who had not had a prior colonoscopy within the preceding 12 months. To approximate the measure denominator (Table 1), we first identified all colonoscopies performed in FY 2017 using Current Procedural Terminology (CPT) and Healthcare Common Procedure Coding System (HCPCS) codes (Table 5). For patients who had more than one colonoscopy performed in FY 2017, only the first (index) procedure was included in the denominator. Likewise, only patients who had no prior colonoscopy performed in the 12 months preceding the index FY2017 colonoscopy were included in the denominator. This is because when a colonoscopy is repeated within a year, there is a high probability that the repeat procedure was done for a reasonable indication such as inadequate bowel preparation on the prior procedure, sedation intolerance leading to an incomplete procedure, or failure to complete the prior procedure due to technical difficulty.

Table 5. Codes used to Identify Colorectal Cancer Screening Tests

**Colonoscopy**	
CPT Codes	44388, 44389, 44390, 44391, 44392, 44393, 44394, 45378, 45379, 45380, 45381, 45382, 45383, 45384, 45385, 45386 (general colonoscopy CPT codes)
HCPCS Codes	G0105, G0121
FOBT LOINC Codes	2335-8, 12503-9, 12504-7, 14563-1, 14564-9, 14565-6, 27396-1, 27401-9, 27925-7, 27926-5, 29771-3, 56490-6, 56491-4, 57905-2, 58453-2

Denominator Exclusions (Exclusion of Non-Average-Risk Screening Colonoscopies): We then excluded procedures that may have been performed for diagnostic or high-risk screening or surveillance indications, using an approach previously developed and validated by Fisher and colleagues.[14] First, we excluded patients who had an ICD-10 code for specific gastrointestinal symptoms or for colorectal neoplasia within 12 months of the FY17 colonoscopy (Table 6).

Table 6. Codes Used to Exclude Colonoscopies Performed for Non-Screening Indications

**ICD-10**	**Description**
D50.0	Iron deficiency anemia secondary to blood loss (chronic)
K50.011	Crohn's disease of small intestine with rectal bleeding
K50.112	Crohn's disease of large intestine with intestinal obstruction
K50.812	Crohn's disease of both small and large intestine with intestinal obstruction
K50.914	Crohn's disease, unspecified, with abscess
K50.919	Crohn's disease, unspecified, with unspecified complications
K51.311	Ulcerative (chronic) rectosigmoiditis with rectal bleeding
K51.313	Ulcerative (chronic) rectosigmoiditis with fistula
K51.318	Ulcerative (chronic) rectosigmoiditis with other complication
K51.319	Ulcerative (chronic) rectosigmoiditis with unspecified complications

K51.418	Inflammatory polyps of colon with other complication
K51.514	Left sided colitis with abscess
K51.813	Other ulcerative colitis with fistula
K51.90	Ulcerative colitis, unspecified, without complications
K52.839	Microscopic colitis, unspecified
K56.0	Paralytic ileus
K57.41	Diverticulitis of both small and large intestine with perforation and abscess with bleeding
K92.2	Gastrointestinal hemorrhage, unspecified
R19.5	Other fecal abnormalities
R63.6	Underweight
K50.119	Crohn's disease of large intestine with unspecified complications
K50.911	Crohn's disease, unspecified, with rectal bleeding
K51.019	Ulcerative (chronic) pancolitis with unspecified complications
K51.212	Ulcerative (chronic) proctitis with intestinal obstruction
K51.811	Other ulcerative colitis with rectal bleeding
K51.911	Ulcerative colitis, unspecified with rectal bleeding
K51.918	Ulcerative colitis, unspecified with other complication
K52.0	Gastroenteritis and colitis due to radiation
K52.89	Other specified noninfective gastroenteritis and colitis
K59.09	Other constipation
K62.89	Other specified diseases of anus and rectum
R15.0	Incomplete defecation
R15.2	Fecal urgency
D50.8	Other iron deficiency anemias
K50.013	Crohn's disease of small intestine with fistula
K50.912	Crohn's disease, unspecified, with intestinal obstruction
K51.00	Ulcerative (chronic) pancolitis without complications
K51.014	Ulcerative (chronic) pancolitis with abscess
K51.211	Ulcerative (chronic) proctitis with rectal bleeding
K51.214	Ulcerative (chronic) proctitis with abscess
K51.412	Inflammatory polyps of colon with intestinal obstruction
K51.814	Other ulcerative colitis with abscess
K51.914	Ulcerative colitis, unspecified with abscess
K56.60	Unspecified Intestinal Obstruction
K57.53	Diverticulitis of both small and large intestine without perforation or abscess with bleeding
K59.01	Slow transit constipation
K59.1	Functional diarrhea
R19.4	Change in bowel habit
R63.4	Abnormal weight loss
K50.019	Crohn's disease of small intestine with unspecified complications
K50.10	Crohn's disease of large intestine without complications
K50.114	Crohn's disease of large intestine with abscess
K50.118	Crohn's disease of large intestine with other complication
K50.819	Crohn's disease of both small and large intestine with unspecified complications
K51.011	Ulcerative (chronic) pancolitis with rectal bleeding

K51.013	Ulcerative (chronic) pancolitis with fistula
K51.213	Ulcerative (chronic) proctitis with fistula
K51.219	Ulcerative (chronic) proctitis with unspecified complications
K51.312	Ulcerative (chronic) rectosigmoiditis with intestinal obstruction
K51.40	Inflammatory polyps of colon without complications
K51.818	Other ulcerative colitis with other complication
K52.9	Noninfective gastroenteritis and colitis, unspecified
K57.33	Diverticulitis of large intestine without perforation or abscess with bleeding
K59.03	Drug induced constipation
K59.04	Chronic idiopathic constipation
K62.5	Hemorrhage of anus and rectum
R15.9	Full incontinence of feces
R19.7	Diarrhea, unspecified
K50.014	Crohn's disease of small intestine with abscess
K50.018	Crohn's disease of small intestine with other complication
K50.80	Crohn's disease of both small and large intestine without complications
K50.811	Crohn's disease of both small and large intestine with rectal bleeding
K50.814	Crohn's disease of both small and large intestine with abscess
K50.918	Crohn's disease, unspecified, with other complication
K51.30	Ulcerative (chronic) rectosigmoiditis without complications
K51.419	Inflammatory polyps of colon with unspecified complications
K51.50	Left sided colitis without complications
K51.519	Left sided colitis with unspecified complications
K51.912	Ulcerative colitis, unspecified with intestinal obstruction
K52.29	Other allergic and dietetic gastroenteritis and colitis
K52.831	Collagenous colitis
K55.21	Angiodysplasia of colon with hemorrhage
K57.21	Diverticulitis of large intestine with perforation and abscess with bleeding
K59.39	Other megacolon
D50.9	Iron deficiency anemia, unspecified
K50.113	Crohn's disease of large intestine with fistula
K50.813	Crohn's disease of both small and large intestine with fistula
K50.818	Crohn's disease of both small and large intestine with other complication
K51.012	Ulcerative (chronic) pancolitis with intestinal obstruction
K51.411	Inflammatory polyps of colon with rectal bleeding
K51.414	Inflammatory polyps of colon with abscess
K51.512	Left sided colitis with intestinal obstruction
K51.80	Other ulcerative colitis without complications
K51.819	Other ulcerative colitis with unspecified complications
K57.93	Diverticulitis of intestine, part unspecified, without perforation or abscess with bleeding
K59.00	Constipation, unspecified
K59.02	Outlet dysfunction constipation
R19.8	Other specified symptoms and signs involving the digestive system and abdomen
R63.0	Anorexia
K50.111	Crohn's disease of large intestine with rectal bleeding

K50.90	Crohn's disease, unspecified, without complications
K51.20	Ulcerative (chronic) proctitis without complications
K51.413	Inflammatory polyps of colon with fistula
K51.511	Left sided colitis with rectal bleeding
K51.913	Ulcerative colitis, unspecified with fistula
K51.919	Ulcerative colitis, unspecified with unspecified complications
K52.832	Lymphocytic colitis
K52.838	Other microscopic colitis
K56.7	Ileus, unspecified
K57.31	Diverticulosis of large intestine without perforation or abscess with bleeding
K57.91	Diverticulosis of intestine, part unspecified, without perforation or abscess with bleeding
K92.1	Melena
R15.1	Fecal smearing
D50.1	Sideropenic dysphagia
K50.00	Crohn's disease of small intestine without complications
K50.012	Crohn's disease of small intestine with intestinal obstruction
K50.913	Crohn's disease, unspecified, with fistula
K51.018	Ulcerative (chronic) pancolitis with other complication
K51.218	Ulcerative (chronic) proctitis with other complication
K51.314	Ulcerative (chronic) rectosigmoiditis with abscess
K51.513	Left sided colitis with fistula
K51.518	Left sided colitis with other complication
K51.812	Other ulcerative colitis with intestinal obstruction
K52.3	Indeterminate colitis
K56.1	Intussusception
K57.51	Diverticulosis of both small and large intestine without perforation or abscess with bleeding
K57.81	Diverticulitis of intestine, part unspecified, with perforation and abscess with bleeding
K92.0	Hematemesis

To further increase the specificity of the electronic measure, we also excluded individuals with ICD-9 and ICD-10 codes indicating high risk for colorectal cancer or prior total abdominal colectomy at any time in the prior 10 years (from FY07 to FY17) (Tables 7a and 7b). Specifically, patients were excluded if CPT or ICD-9/-10 codes revealed any of the following diagnoses between FY07 and the qualifying FY17 colonoscopy: (1) prior colectomy, (2) history of colorectal cancer; (3) history of colon polyps; (4) history of inflammatory bowel disease; or (5) family history of colorectal cancer. Both ICD-9 and ICD-10 codes were used because VHA (like most US healthcare systems) transitioned between these two coding systems in October 2015. These additional exclusion criteria were selected to ensure that the cohort comprised individuals who were at average (rather than increased) risk of CRC. Finally, we excluded individuals who underwent their FY17 colonoscopy during a hospitalization (since such colonoscopies are unlikely to be performed for screening). Thus, the final denominator (after all exclusions) consisted of all average-risk screening colonoscopies performed in FY2017. The sensitivity of the ICD-10-based electronic measure for screening indication was 79%, compared to 36% for the ICD-9-based measure.

Table 7a. Additional Codes Used to Identify Patients at Increased Risk for Colorectal Cancer (ICD-9)

**Diagnosis**	**Code**
Colectomy (CPT)	44150 - 44153, 44155, 44156, 44210 - 44212
Colorectal cancer (ICD-9)	153.×, 154.0, 154.1, 154.8, V10.0, V10.05, V10.06
Colorectal cancer (HCPCS)	G0105, G0213, G0214, G0215, G0231
Colon polyps (ICD-9)	211.3, 211.4, 230.3, 230.4, V12.72
Inflammatory bowel disease (ICD-9)	555.×, 556.x
Family history of colorectal cancer (ICD-9)	V16.0, V18.51

CPT = Current Procedural Terminology; ICD-10 = International Classification of Disease, Tenth Revision; HCPCS = Healthcare Common Procedure Coding System

Table 7b. Additional Codes Used to Identify Patients at Increased Risk for Colorectal Cancer (ICD-10)

**Diagnosis**	**Code**
Colectomy (CPT)	44150 - 44153, 44155, 44156, 44210 - 44212
Colorectal cancer (ICD-10)	C18.0, C18.1, C18.2, C18.3, C18.4, C18.5, C18.6, C18.7, C18.8, C18.9, C19., C20., C21.2, C21.8, Z85.00, Z85.01, Z85.028, Z85.038, Z85.048, Z85.05, Z85.068, Z85.07, Z85.09, Z85.810, Z85.818, Z85.819
Colorectal cancer (HCPCS)	G0105, G0213, G0214, G0215, G0231
Colon polyps (ICD-10)	D01.0, D01.1, D01.2, D12.0, D12.1, D12.2, D12.3, D12.4, D12.5, D12.6, D12.7, D12.8, D12.9, K63.5, Z86.010
Inflammatory bowel disease (ICD-10)	K50.00, K50.10, K50.80, K50.90, K51.80, K51.20, K51.30, K51.40, K51.50, K51.00, K51.90
Family history of colorectal cancer (ICD-10)	Z80.0, Z83.71

CPT = Current Procedural Terminology; ICD-10 = International Classification of Disease, Tenth Revision; HCPCS = Healthcare Common Procedure Coding System

Electronic Approximation of Measure Numerator

Specification of electronic elements comprising the measure numerator (probable and possible screening colonoscopy overuse – Table 1) was more straightforward than for the denominator since these elements were primarily based on factors such as patient age and the time interval between colonoscopies that are reliably-coded in administrative data. To identify fecal occult blood tests (FOBTs), we used Logical Observation Identifiers Names and Codes (LOINC) (Table 5). To identify patients with life expectancy <6 months, we used structured data from CDW that is used to indicate limited life expectancy for clinical purposes.
