# Supplementary material for: Development and validation of a new ICD-10-based screening colonoscopy overuse measure in a large integrated healthcare system: a retrospective observational study
Source: BMJ Qual Saf. Author manuscript; Available in PMC 2023 Jul 10. (PMC10294020; doi:10.1136/bmjqs-2021-014236)
Supplement: Supp2 — Appendix 2. Abstraction instrument for manual record review. [file NIHMS1894124-supplement-Supp2.pdf]

Appendix 2. Abstraction instrument for manual record review

VHA EPRP
COLORECTAL CANCER SCREENING COLONOSCOPY OVERUSE STUDY_v3
FY2019

#	Name	QUESTION	Field Format	DEFINITIONS/DECISION RULES
		**Organizational Identifiers**		
	VAMC<br>CONTROL<br>QIC<br>BEGDTE<br>REVDTE	Facility ID<br>Control Number<br>Abstractor ID<br>Abstraction Begin Date<br>Abstraction End Date	Auto-fill<br>Auto-fill<br>Auto-fill<br>Auto-fill<br>Auto-fill	
		**Patient Identifiers**		
	SSN<br>PTNAMEF<br>PTNAMEL<br>BIRTHDT<br>SEX<br>MARISTAT<br>RACE	Patient SSN<br>First Name<br>Last Name<br>Birth Date<br>Sex<br>Marital Status<br>Race	Auto-fill: no change<br>Auto-fill: no change<br>Auto-fill: no change<br>Auto-fill: no change<br>Auto-fill: can change<br>Auto-fill: no change<br>Auto-fill: no change	
		**Index Colonoscopy**		
1	indexdt	Enter the date of the index colonoscopy completed during the timeframe from 1/01/2017 through 12/31/2017.	mm/dd/yyyy<br>**Computer will pre-fill from pull list**<br>>= 01/01/2017 and <= 12/31/2017	Computer will pre-fill the date of the index colonoscopy completed in during the specified timeframe.
2	indxcol	On (computer to display indexdt), is there documentation of the index colonoscopy in the medical record?<br>1. Yes<br>2. No	1,2<br>If 1, auto-fill othindcol as 95, othcoldt as 99/99/9999, and go to colinpt; else go to othindcol	Search procedure reports, surgical reports, and progress notes for documentation that a colonoscopy was performed on the date entered in INDEXDT.

Appendix 2. Abstraction instrument for manual record review

VHA EPRP
COLORECTAL CANCER SCREENING COLONOSCOPY OVERUSE STUDY_v3
FY2019

#	Name	QUESTION	Field Format	DEFINITIONS/DECISION RULES
3	othindcol	During the timeframe from (computer to display indexdt – 180 days to indexdt + 180 days), is there documentation a colonoscopy was performed?<br>1. Yes<br>2. No<br>95. Not applicable	1,2,95<br>Will be auto-filled as 95 if indxcol = 1<br>***If 2, go to pretac**	Search procedure reports, surgical reports, and progress notes for documentation that a colonoscopy was performed within 180 days prior to or after the pre-filled index colonoscopy date.
4	othcoldt	Enter the date of the most recent colonoscopy completed during the timeframe from (computer to display indexdt – 180 days to indexdt + 180 days).	mm/dd/yyyy<br>Will be auto-filled as 99/99/9999 if indxcol = 1<br><= 180 days prior to indexdt and <= 180 days after indexdt and < = stdyend	If more than one colonoscopy was performed during the timeframe, enter the date of the most recent colonoscopy.
5	colinpt	Was the index colonoscopy performed during an inpatient hospitalization at a VAMC?<br>1. Yes<br>2. No	1,2	If the index colonoscopy was performed during an inpatient hospitalization at any VAMC, answer "1". If the colonoscopy was performed as an outpatient procedure prior to an inpatient admission, answer "2." For example, patient is scheduled for outpatient colonoscopy and has a complication during the procedure requiring admission; answer "2."
6	colnrpt	Was the colonoscopy procedure report/note found in the medical record?<br>3. Procedure report found in CPRS<br>4. Procedure report found in VISTA imaging<br>99. Procedure report not found in medical record	3,4,99<br>**If 99, go to pretac**<br>**Warning if 99 and colinpt = 1**	If the actual colonoscopy report/note is not found in CPRS or VISTA imaging, enter "99".<br>Suggested data sources: Notes, consults, procedures, VISTA imaging
7	colnrp_txt	Enter the note title of the colonoscopy procedure report.	Text<br>(limit 150 characters)	Enter the local note title as documented in the record. Examples include, but are not limited to:<br>Local Title: Colonoscopy Procedure Note<br>Local Title: Co-GI Consult Procedure Note

Appendix 2. Abstraction instrument for manual record review

VHA EPRP
COLORECTAL CANCER SCREENING COLONOSCOPY OVERUSE STUDY_v3
FY2019

#	Name	QUESTION	Field Format	DEFINITIONS/DECISION RULES
8	anescoln	Is there documentation that an anesthesiologist or Certified Registered Nurse Anesthetist (CRNA) was present at the time of the index colonoscopy to sedate the patient for the procedure?<br>1. Yes<br>2. No	1,2	**The intent of this question is to determine if the patient was sedated for the procedure under the supervision of an anesthesiologist or Certified Registered Nurse Anesthetist (CRNA).** This type of procedural sedation may be documented as Monitored Anesthesia Care (MAC).<br>Look at the colonoscopy report and any peri-procedural notes (including sedation records) available in the medical record for documentation of procedural sedation.<br>**Note:** may see the terms "anesthesia," "anesthesiologist," "CRNA (Certified Registered Nurse Anesthetist)", "Propofol" (under medications), "See anesthesia note," or "MAC (monitored anesthesia care)."<br>If any of the above terms are documented, select "Yes."<br>If none of these terms are present, select "No."
9	colind1	Is there physician/APN/PA documentation of a specific reason (indication) the index colonoscopy was performed?<br>1. Yes<br>2. No	1,2<br>If 2, auto-fill crcscrn as 95, highrsk as 95, nonscrn as 95, and go to anatext	**Only Acceptable Source: Colonoscopy Report or Colonoscopy Procedure Note**<br>Other terms for reason (indication) may be used (e.g., pre-operative diagnosis).<br>Reasons (indication) for performing a colonoscopy may include, but are not limited to:<br>● Family history of colorectal cancer<br>● Family history of colorectal adenomas or polyps<br>● Personal history of polyps<br>● Personal history of colorectal cancer<br>● Inflammatory bowel disease, Crohn's disease, or ulcerative colitis<br>● High risk, NOS<br>● Screening only<br>● Surveillance, NOS<br>● Symptoms such as bleeding, change in bowel habits, abdominal pain

Appendix 2. Abstraction instrument for manual record review

#	Name	QUESTION	Field Format	DEFINITIONS/DECISION RULES
10	crcscrn	Is there physician/APN/PA documentation the index colonoscopy was performed for average-risk (routine) screening only?<br>1. Yes<br>2. No<br>95. Not applicable	1,2,95<br>Will be auto-filled as 95 if colind1 = 2<br>If 1, go to anatext	**Only Acceptable Source: Colonoscopy Report or Colonoscopy Procedure Note**<br>In order to answer "1", the physician/APN/PA documentation must clearly indicate the colonoscopy was performed for average-risk screening only. It is not necessary to see the term "average-risk".<br>If documentation indicates the colonoscopy was performed for routine screening, select "1".<br>If documentation indicates the colonoscopy was performed due to symptoms such as bleeding, change in bowel habits, abdominal pain or any of the high-risk screening/surveillance indications (e.g., family or personal history colorectal cancer, family or personal history polyps, inflammatory bowel disease, Crohn's disease, ulcerative colitis, high risk, or surveillance), enter "2".<br>**If the indication documented in the colonoscopy report/procedure note is conflicting (i.e., average-risk screening and rectal bleeding), enter "2".**
11	highrsk1<br>highrsk2<br>highrsk3<br>highrsk4<br>highrsk5<br>highrsk6<br>highrsk7<br>highrsk95<br>highrsk99	Select all high-risk screening or surveillance reasons/indications documented in the colonoscopy report/procedure note.<br>**Indicate all that apply:**<br>1. Family history of colorectal cancer<br>2. Family history of colorectal adenomas or polyps<br>3. Personal history of polyps<br>4. Personal history of colorectal cancer<br>5. Inflammatory bowel disease, Crohn's disease, or ulcerative colitis<br>6. High risk, NOS<br>7. Surveillance, NOS<br>95. Not applicable<br>99. None of the above	1,2,3,4,5,6,7,95,99<br>Will be auto-filled as 95 if colind1 = 2	**Only Acceptable Source: Colonoscopy Report or Colonoscopy Procedure Note**<br>Other terms for reason may be used (e.g., pre-operative diagnosis).<br>High-risk screening or surveillance involves testing patients that previously had colorectal cancer or are at increased risk for colorectal cancer.

#	Name	QUESTION	Field Format	DEFINITIONS/DECISION RULES
12	nonscrn	Is there physician/APN/PA documentation that the index colonoscopy was performed for a non-screening diagnostic indication (bleeding, positive FOBT, anemia, diarrhea, constipation, abdominal pain, etc.)?<br>1. Yes<br>2. No<br>95. Not applicable	1,2,95<br>Will be auto-filled as 95 if colind1 = 2<br>Hard edit: If highrsk99 = -1 cannot = 2	**Only Acceptable Source: Colonoscopy Report or Colonoscopy Procedure Note**<br>Other terms for reason may be used (e.g., pre-operative diagnosis).<br>**Non-screening diagnostic indication** = person has symptoms of a condition or disease.<br>If any non-screening diagnostic indication is documented by the physician/APN/PA as the reason for performing the colonoscopy, answer "1".
13	anatext	Is there documentation of the anatomic **extent reached** during the index colonoscopy?<br>1. Yes<br>2. No	1,2<br>If 2, auto-fill anareach as 95, and go to qualprep	**Only Acceptable Source: Colonoscopy Report or Colonoscopy Procedure Note**<br>Anatomic extent reached may be documented under a heading (e.g., **Extent of Examination:** Cecum) or in body of procedure report/note. For example:<br>**Description of Procedure and Findings:** Informed consent was obtained. Rectal examination by finger was unremarkable. The scope was introduced all the way up to the cecum without any difficulty.
14	anareach	Is there documentation the cecum, appendix, appendiceal orifice, ileum, or small bowel was reached during the index colonoscopy?<br>1. Yes<br>2. No<br>95. Not applicable	1,2,95<br>Will be auto-filled as 95 if anatext = 2	**Only Acceptable Source: Colonoscopy Report or Colonoscopy Procedure Note**<br>If there is documentation that the cecum, appendix, appendiceal orifice, ileum, or small bowel was reached during the colonoscopy, answer "1".

Appendix 2. Abstraction instrument for manual record review

#	Name	QUESTION	Field Format	DEFINITIONS/DECISION RULES
15	qualprep	Is there documentation of the **quality of bowel preparation** in the index colonoscopy report?<br>1. Yes<br>2. No	1,2<br>If 2, auto-fill poorprep as 95, prep as 95, and go to bbpsprep	**Only Acceptable Source: Colonoscopy Report or Colonoscopy Procedure Note**<br>Quality of preparation may be documented under a heading or in the body of the procedure report/note. In order to answer "yes", the header or statement must clearly refer to the overall quality of the bowel preparation. Disregard statements about visual quality as the scope is advanced through different sections of the colon that occur in the body rather than header of the report (i.e., are descriptors of the endoscopy rather than a summary assessment of overall preparation quality).<br>**Examples include but are not limited to:**<br>"Parts of the transverse colon were obscured by stool" or "The cecum was adequate after extensive cleaning."<br>However, the endoscopist may write an overall prep assessment such as "The quality of the preparation was excellent except the cecum was poor" which is acceptable.<br>**Exclude:** Documentation of Boston Bowel Prep Scale
16	poorprep	Is there any mention of the bowel preparation being suboptimal, poor, or inadequate on the index colonoscopy?<br>1. Yes<br>2. No<br>95. Not applicable	1,2,95<br>Will be auto-filled as 95 if qualprep = 2	**Only Acceptable Source: Colonoscopy Report or Colonoscopy Procedure Note**<br>If there is any mention that the quality of bowel preparation was suboptimal, poor, or inadequate (or similar term indicating the prep was unsatisfactory), answer "1".

#	Name	QUESTION	Field Format	DEFINITIONS/DECISION RULES
17	prep1<br>prep2<br>prep3<br>prep4<br>prep5<br>prep95<br>prep99	Indicate the quality of bowel preparation documented in the procedure report/note.<br>**Indicate all that apply:**<br>1. Adequate (or satisfactory)<br>2. Good (or excellent)<br>3. Fair<br>4. Inadequate (or unsatisfactory)<br>5. Suboptimal (or poor)<br>95. Not applicable<br>99. None of the above or unable to determine	1,2,3,4,5,95,99<br>Will be auto-filled as 95 if qualprep = 2<br>Cannot enter 99 with any other number<br>Cannot enter 1 or 2 with 3, 4, or 5	**Only Acceptable Source: Colonoscopy Report or Colonoscopy Procedure Note**<br>Select the value(s) that indicate the quality of bowel preparation documented in the procedure report/note. If the term used to describe the quality of bowel preparation is not listed or is not similar to one of the terms listed, select “99”.<br>If bowel prep quality documentation is conflicting (e.g. adequate except cecum was poor), select “99”.
18	bbpsprep	Was the Boston Bowel Preparation Scale (BBPS) used to document the quality of bowel preparation?<br>1. Yes<br>2. No	1,2<br>If 2, go to indbiop	**Only Acceptable Source: Colonoscopy Report or Colonoscopy Procedure Note**<br>The BBPS requires a score be assigned to each segment (right (cecum and ascending), transverse, left (descending, sigmoid and rectum)) of the colon. The segment scores range from 0 – 3 and are summed for a total BBPS score ranging from 0 -9. Segment points are assigned by the endoscopist as follows:<br>0 = unprepared colon segment with mucosa note seen due to solid stool that cannot be cleared<br>1 = Portion of mucosa of the colon segment seen, but other areas of the colon segment not well seen due to staining, residual stool and/or opaque liquid.<br>2 = Minor amount of residual staining, small fragments of stool and/or opaque liquid, but mucosa of colon segment seen well.<br>3 = Entire mucosa of colon segment seen well with no residual staining, small fragments of stool or opaque liquid.<br><br>If there is documentation the BBPS was used to document the quality of bowel preparation, select “1”.

#	Name	QUESTION	Field Format	DEFINITIONS/DECISION RULES
19	bbpsr<br>bbpst<br>bbpsd	Enter the BBPS score for each colon segment documented in the colonoscopy report/procedure note.<br>**Colon Segment** / **Score** (Whole numbers 0 – 3, Abstractor may enter zz)<br>1. Right colon (ascending) / 2. Transverse colon / 3. Left colon (descending) /		**Only Acceptable Source: Colonoscopy Report or Colonoscopy Procedure Note**<br>**Enter the BBPS score for each colon segment documented in the colonoscopy report/procedure note.**<br>If the score for a colon segment is not documented enter zz.
20	bbpstot	Enter the total BBPS score documented in the colonoscopy report/procedure note.	Whole numbers<br>0 -9<br>May enter zz	**Only Acceptable Source: Colonoscopy Report or Colonoscopy Procedure Note**<br>Enter the BBPS total score documented in the colonoscopy report/procedure note.<br>If the total BBPS score is not documented in the colonoscopy report/procedure note, enter zz.
21	indbiop	Was a biopsy or other tissue removal (e.g., polypectomy) performed during the index colonoscopy (computer to display indexdt?<br>1. Yes<br>2. No<br>99. Unable to determine	1,2<br>If 2 or 99, auto-fill indbiop as 95,and go to colnordt	**Review the colonoscopy procedure note/report to determine if a biopsy or other tissue removal (polypectomy) was performed during the procedure.**<br>If the colonoscopy report/procedure note is in the record and there is documentation a biopsy or other tissue removal (polypectomy) was performed, enter “1”.<br>If the colonoscopy report/procedure note is in the record and there is no documentation a biopsy or other tissue removal (polypectomy) was performed, enter “2”.<br>If the colonoscopy report is not available in the record and no other documentation indicates if a biopsy or other tissue removal (e.g., polypectomy) was performed, enter “99”.<br>Suggested data sources (CPRS and scanned documents): Colonoscopy report, Colonoscopy procedure note

Appendix 2. Abstraction instrument for manual record review

VHA EPRP
COLORECTAL CANCER SCREENING COLONOSCOPY OVERUSE STUDY_v3
FY2019

#	Name	QUESTION	Field Format	DEFINITIONS/DECISION RULES
22	indbiop1<br>indbiop2<br>indbiop3<br>indbiop95<br>indbiop99	Do pathology results from biopsy/tissue removal show any of the following?<br>Indicate all that apply:<br>1. Adenoma or adenomatous polyp of the colon or rectum<br>2. Cancer, carcinoma, or adenocarcinoma of the colon or rectum<br>3. Inflammatory bowel disease (IBD, ulcerative colitis, or Crohn's disease)<br>95. Not applicable<br>99. None of the above	1,2,3,95,99<br>Will be auto-filled as 95 if indbiop = 2 or 99	**Suggested data source: Pathology report**

#	Name	QUESTION	Field Format	DEFINITIONS/DECISION RULES
23	colnordt	For the colonoscopy performed on (computer display indexdt), enter the earliest date the index colonoscopy was ordered by the physician/APN/PA.	mm/dd/yyyy<br>Abstractor may enter 99/99/9999<br><= 1 year prior indexdt and < indexdt	Start by looking at the physician orders for the index colonoscopy. If documentation is available, enter the earliest date the colonoscopy was ordered. If no information is available in the physician orders, review the consult request and clinic notes to determine the date the colonoscopy was requested by the physician/APN/PA.<br>Enter the earliest date the index colonoscopy was ordered by the physician/APN/PA.<br>Suggested data sources: consult request, clinic notes, physician orders
24	ordind	Is there documentation by the referring physician/APN/PA of a specific reason (indication) the index colonoscopy was ordered?<br>1. Yes<br>2. No	1,2<br>If 2, auto-fill crcscrn2 as 95, ordrsk as 95, ordiag as 95, and go to lifexpec	Look for documentation of a specific reason (indication) the colonoscopy was ordered in the progress notes (or consult request) of the physician/APN/PA that referred the patient for the colonoscopy.<br>**Reasons (indication) for performing a colonoscopy may include, but are not limited to:**<br>● Family history of colorectal cancer<br>● Family history of colorectal adenomas or polyps<br>● Personal history of polyps<br>● Personal history of colorectal cancer<br>● Inflammatory bowel disease, Crohn’s disease, or ulcerative colitis<br>● High risk, NOS<br>● Surveillance, NOS<br>● Symptoms such as bleeding, change in bowel habits, abdominal pain<br>● Screening only

#	Name	QUESTION	Field Format	DEFINITIONS/DECISION RULES
25	crcscrn2	Is there documentation by the referring physician/APN/PA that the index colonoscopy was ordered for average-risk (routine) screening only?<br>1. Yes<br>2. No<br>95. Not applicable	1,2,95<br>Will be auto-filled as 95 if ordind = 2<br>If 1, go to lifexpec	In order to answer "1", the physician/APN/PA that referred the patient for the colonoscopy must clearly indicate the colonoscopy was ordered for average-risk screening only.<br>It is not necessary to see the term "average-risk".<br>If documentation indicates the colonoscopy was ordered for routine screening, select "1".<br>If documentation indicates the colonoscopy was ordered due to symptoms such as bleeding, change in bowel habits, abdominal pain or any of the high-risk screening/surveillance indications listed below, enter "2".<br>**High Risk Screening/Surveillance Indications:**<br>● Family history of colorectal cancer<br>● Family history of colorectal adenomas or polyps<br>● Personal history of polyps<br>● Personal history of colorectal cancer<br>● Inflammatory bowel disease, Crohn's disease, or ulcerative colitis<br>● High risk, NOS<br>● Surveillance, NOS

Appendix 2. Abstraction instrument for manual record review

#	Name	QUESTION	Field Format	DEFINITIONS/DECISION RULES
26	ordrsk1<br>ordrsk2<br>ordrsk3<br>ordrsk4<br>ordrsk5<br>ordrsk6<br>ordrsk7<br>ordrsk95<br>ordrsk99	Select all high-risk screening or surveillance indications documented by the ordering physician/APN/PA.<br>Indicate all that apply:<br>1. Family history of colorectal cancer<br>2. Family history of colorectal adenomas or polyps<br>3. Personal history of polyps<br>4. Personal history of colorectal cancer<br>5. Inflammatory bowel disease, Crohn's disease, or ulcerative colitis<br>6. High risk, NOS<br>7. Surveillance, NOS<br>95. Not applicable<br>99. None of the above	1,2,3,4,5,6,7,95,99<br>Will be auto-filled as 95 if ordind = 2	Other terms for reason may be used (e.g., pre-operative diagnosis). High-risk screening or surveillance involves testing patients that previously had colorectal cancer or are at increased risk for colorectal cancer.<br>**Reasons (indication) for ordering a colonoscopy may include, but are not limited to:**<br>● Family history of colorectal cancer<br>● Family history of colorectal adenomas or polyps<br>● Personal history of polyps Personal history of colorectal cancer<br>● Inflammatory bowel disease, Crohn's disease, or ulcerative colitis<br>● High risk, NOS<br>● Surveillance, NOS
27	ordiag	Was the index colonoscopy ordered for a non-screening diagnostic indication (bleeding, anemia, positive FOBT, diarrhea, constipation, abdominal pain, etc.)?<br>1. Yes<br>2. No<br>95. Not applicable	1,2,95<br>Will be auto-filled as 95 if ordind = 2<br>If 1 or 2, go to lifexpec<br>Hard edit: If ordrsk99 = -1 cannot = 2	**Non-screening diagnostic indication = person has symptoms of a condition or disease**<br>**If any non-screening diagnostic indication is documented by the physician/APN/PA as the reason for ordering the colonoscopy, answer "1".**

Appendix 2. Abstraction instrument for manual record review

#	Name	QUESTION	Field Format	DEFINITIONS/DECISION RULES
28	pretac	Prior to the date of the index colonoscopy on (computer to display indexdt), is there evidence in the record that the patient had a total abdominal colectomy?<br>1. Yes<br>2. No	1,2	**Total abdominal colectomy** = Complete removal of the colon including the rectum
29	lifexpec	During CY2016 or CY2017, is there documentation that the patient's life expectancy is less than 6 months?<br>1. Yes<br>2. No	1,2	Patient's life expectancy of less than six months must be documented on the problem list or in the computer field "health factors," without exception.
		**Non-Index Colonoscopy**		
30	nonindt1	Enter the date of the most recent non-index colonoscopy performed prior to the index colonoscopy.	Mm/dd/yyyy<br>**Computer will pre-fill date of most recent non-index colonoscopy**<br>***If null, go to colfobt**<br><= 10 years prior to indexdt and < indexdt	Computer will pre-fill the date of the most recent non-index colonoscopy performed within 10 years prior to the index colonoscopy.
31	noncol1	On (computer to display nonindt1), is there documentation that the non-index colonoscopy was performed?<br>1. Yes<br>2. No	1,2<br>If 1, auto-fill othnon1 as 95, actnondt1 as 99/99/9999, and go to pcolnva4	**Non-index colonoscopy** = colonoscopy performed prior to the index colonoscopy<br>Search procedure reports, surgical reports, and progress notes for documentation that a non-index colonoscopy was performed on the date entered in NONINDT1. Reports may also be found in VISTA Imaging.

Appendix 2. Abstraction instrument for manual record review

#	Name	QUESTION	Field Format	DEFINITIONS/DECISION RULES
32	othnon1	During the timeframe from (computer to display nonindt1 – 180 days to nonindt1 + 180 days), is there documentation a non-index colonoscopy was performed?<br>1. Yes<br>2. No<br>95. Not applicable	1,*2,95<br>Will be auto-filled as 95 if noncol1= 1<br>***If 2, go to colfobt**	Search procedure reports, surgical reports, and progress notes for documentation that a non-index colonoscopy was performed within 180 days prior to or after the pre-filled non-index colonoscopy date. Reports may also be found in VISTA Imaging.
33	actnondt1	Enter the date of the most recent non-index colonoscopy completed during the timeframe from (computer to display nonindt1 – 180 days to nonindt1 + 180 days).	mm/dd/yyyy<br>Will be auto-filled as 99/99/9999 if noncol1 = 1<br><= 180 days prior to or after nonindt1	If more than one non-index colonoscopy was performed within 180 days prior to or after the pre-filled non-index colonoscopy date, enter the date of colonoscopy performed most immediately prior to the index colonoscopy.
34	pcolnva4	Was the non-index colonoscopy performed at a VAMC?<br>1. Yes<br>2. No	1,*2<br>*If 2, go to nonindt2	If the non-index colonoscopy was performed at a VAMC, enter "1".<br>If the non-index colonoscopy was performed outside the VHA, enter "2".
35	noninpt1	Was the non-index colonoscopy performed during an inpatient hospitalization at a VAMC?<br>1. Yes<br>2. No	1,2	If the non-index colonoscopy was performed during an inpatient hospitalization at any VAMC, answer "1".<br>If the non-index colonoscopy was performed as an outpatient procedure prior to an inpatient admission, answer "2." For example, patient is scheduled for outpatient colonoscopy and has a complication during the procedure requiring admission; answer "2."
36	colnrpt2	Was the non-index colonoscopy procedure report/note found in the medical record?<br>3. Procedure report found in CPRS<br>4. Procedure report found in VISTA imaging<br>99. Procedure report not found in medical record	3,4,99<br>If 99, go to nonindt2 as applicable<br>**Warning if 99 and pcolnva4 or noninpt1 = 1**	If the actual non-index colonoscopy report is not found in CPRS or VISTA imaging, enter "99".<br>Suggested data sources: Notes, consults, procedures, VISTA imaging

#	Name	QUESTION	Field Format	DEFINITIONS/DECISION RULES
37	ncolnrp_txt	Enter the note title of the non-index colonoscopy procedure report.	Text<br>(limit 150 characters)	Enter the local note title as documented in the record. Examples include, but are not limited to:<br>Local Title: Colonoscopy Procedure Note<br>Local Title: Co-GI Consult Procedure Note
38	anescoln2	Is there documentation that an anesthesiologist or Certified Registered Nurse Anesthetist (CRNA) was present at the time of the non-index colonoscopy to sedate the patient for the procedure?<br>1. Yes<br>2. No	1,2	The intent of this question is to determine if the patient was sedated for the procedure under the supervision of an anesthesiologist or Certified Registered Nurse Anesthetist (CRNA). This type of procedural sedation may be documented as Monitored Anesthesia Care (MAC).<br>Look at the colonoscopy report and any peri-procedural notes (including sedation records) available in the medical record for documentation of procedural sedation.<br>**Note:** may see the terms "anesthesia," "anesthesiologist," "CRNA (Certified Registered Nurse Anesthetist)", "Propofol" (under medications), "See anesthesia note," or "MAC (monitored anesthesia care)."<br>If any of the above terms are documented, select "Yes."<br>If none of these terms are present, select "No."

#	Name	QUESTION	Field Format	DEFINITIONS/DECISION RULES
39	nonindx1	Is there documentation of a specific reason (indication) the non-index colonoscopy was performed?<br>1. Yes<br>2. No	1,2<br>If 2, auto-fill crcscrn3 as 95, nonrsk as 95, nindscr as 95, and go to nordind	**Only Acceptable Source: Colonoscopy Report or Colonoscopy Procedure Note**<br>Other terms for reason (indication) may be used (e.g., pre-operative diagnosis).<br>Reasons (indication) for performing a colonoscopy may include, but are not limited to:<br>● Family history of colorectal cancer<br>● Family history of colorectal adenomas or polyps<br>● Personal history of polyps<br>● Personal history of colorectal cancer<br>● Inflammatory bowel disease, Crohn's disease, or ulcerative colitis<br>● High risk, NOS<br>● Surveillance, NOS<br>● Symptoms such as bleeding, change in bowel habits, abdominal pain<br>● Screening only

Appendix 2. Abstraction instrument for manual record review

VHA EPRP
COLORECTAL CANCER SCREENING COLONOSCOPY OVERUSE STUDY_v3
FY2019

#	Name	QUESTION	Field Format	DEFINITIONS/DECISION RULES
40	crcscrn3	Is there physician/APN/PA documentation the non-index colonoscopy was performed for average-risk (routine) screening only?<br>1. Yes<br>2. No<br>95. Not applicable	1,2,95<br>Will be auto-filled as 95 if nonindx1 = 2<br>If 1, go to nordind	**Only Acceptable Source: Colonoscopy Report or Colonoscopy Procedure Note**<br>In order to answer "1", the physician/APN/PA documentation must clearly indicate the colonoscopy was performed for average-risk screening only.<br>It is not necessary to see the term "average-risk".<br>If documentation indicates the colonoscopy was performed for routine screening, select "1".<br>If documentation indicates the colonoscopy was performed due to symptoms such as bleeding, change in bowel habits, abdominal pain or any of the high-risk screening/surveillance indications listed below, enter "2".<br>**High Risk Screening/Surveillance Indications:**<br>● Family history of colorectal cancer<br>● Family history of colorectal adenomas or polyps<br>● Personal history of polyps<br>● Personal history of colorectal cancer<br>● Inflammatory bowel disease, Crohn's disease, or ulcerative colitis<br>● High risk, NOS<br>● Surveillance, NOS<br>**If the indication documented in the colonoscopy report/procedure note is conflicting (i.e., average-risk screening and rectal bleeding), enter "2".**

Appendix 2. Abstraction instrument for manual record review

VHA EPRP
COLORECTAL CANCER SCREENING COLONOSCOPY OVERUSE STUDY_v3
FY2019

#	Name	QUESTION	Field Format	DEFINITIONS/DECISION RULES
41	nonrsk1<br>nonrsk2<br>nonrsk3<br>nonrsk4<br>nonrsk5<br>nonrsk6<br>nonrsk7<br>nonrsk95<br>nonrsk99	Select all high-risk screening or surveillance indications documented in the non-index colonoscopy report/procedure note.<br>**Indicate all that apply:**<br>1. Family history of colorectal cancer<br>2. Family history of colorectal adenomas or polyps<br>3. Personal history of polyps<br>4. Personal history of colorectal cancer<br>5. Inflammatory bowel disease, Crohn's disease, or ulcerative colitis<br>6. High risk, NOS<br>7. Surveillance, NOS<br>95. Not applicable<br>99. None of the above	1,2,3,4,5,6,7,95,99<br>Will be auto-filled as 95 if nonindx1 = 2	**Only Acceptable Source: Colonoscopy Report or Colonoscopy Procedure Note**<br>Other terms for reason may be used (e.g., pre-operative diagnosis).<br>High-risk screening or surveillance involves testing patients that previously had colorectal cancer or are at increased risk for colorectal cancer.
42	nindscn	Is there physician/APN/PA documentation the non-index colonoscopy performed for a non-screening diagnostic indication (bleeding, positive FOBT, anemia, diarrhea, constipation, abdominal pain, etc.)?<br>1. Yes<br>2. No<br>95. Not applicable	1,2<br>Will be auto-filled as 95 if nonindx1 = 2<br>Hard edit: If nonrsk99 = -1 cannot = 2	**Only Acceptable Source: Colonoscopy Report or Colonoscopy Procedure Note**<br>Other terms for reason may be used (e.g., pre-operative diagnosis).<br>**Non-screening diagnostic indication** = person has symptoms of a condition or disease<br>If any non-screening diagnostic indication is documented by the physician/APN/PA as the reason for performing the colonoscopy, answer "1".
43	nanatext	Is there documentation of the anatomic extent reached during the non-index colonoscopy?<br>1. Yes<br>2. No	1,2<br>If 2, auto-fill nanreach as 95, and go to nqualprep	**Only Acceptable Source: Colonoscopy Report or Colonoscopy Procedure Note**<br>Anatomic extent reached may be documented under a heading (e.g., **Extent of Examination:** Cecum) or in body of procedure report/note. For example:<br>**Description of Procedure and Findings:** Informed consent was obtained. Rectal examination by finger was unremarkable. **The scope was introduced all the way up to the cecum without any difficulty.**

#	Name	QUESTION	Field Format	DEFINITIONS/DECISION RULES
44	nanreach	Is there documentation the cecum, appendix, appendiceal orifice, ileum, or small bowel was reached during the non-index colonoscopy?<br>1. Yes<br>2. No<br>95. Not applicable	1,2,95<br>Will be auto-filled as 95 if nanatext = 2	**Only Acceptable Source: Colonoscopy Report or Colonoscopy Procedure Note**<br>If there is documentation that the cecum, appendix, appendiceal orifice, ileum, or small bowel was reached during the colonoscopy, answer "1".
45	nqualprep	Is there documentation of the quality of bowel preparation on the non-index colonoscopy?<br>1. Yes<br>2. No	1,2<br>If 2, auto-fill npoorprep as 95, nprep as 95, and go to nbbpsprep	**Only Acceptable Source: Colonoscopy Report or Colonoscopy Procedure Note**<br>Quality of preparation may be documented under a heading or in the body of the procedure report/note.<br>In order to answer "yes", the header or statement must clearly refer to the overall quality of the bowel preparation. Disregard statements about visual quality as the scope is advanced through different sections of the colon that occur in the body rather than header of the report (i.e., are descriptors of the endoscopy rather than a summary assessment of overall preparation quality). **Examples include but are not limited to:**<br>"Parts of the transverse colon were obscured by stool" or "The cecum was adequate after extensive cleaning."<br>However, the endoscopist may write an overall prep assessment such as "The quality of the preparation was excellent except the cecum was poor" which is acceptable.<br>**Exclude:** Documentation of Boston Bowel Prep Scale
46	npoorprep	Is there any mention of the bowel preparation being suboptimal, poor, or inadequate on the non-index colonoscopy?<br>1. Yes<br>2. No<br>95. Not applicable	1,2,95<br>Will be auto-filled as 95 if nqualprep = 2	**Only Acceptable Source: Colonoscopy Report or Colonoscopy Procedure Note**<br>If there is any mention that the quality of bowel preparation was suboptimal, poor, or inadequate, answer "1". If quality of bowel prep is described as satisfactory (or other similar term), answer "2".

#	Name	QUESTION	Field Format	DEFINITIONS/DECISION RULES
47	nprep1<br>nprep2<br>nprep3<br>nprep4<br>nprep5<br>nprep95<br>nprep99	Indicate the quality of bowel preparation documented in the procedure report/note.<br>**Indicate all that apply:**<br>1. Adequate (or satisfactory)<br>2. Good (or excellent)<br>3. Fair<br>4. Inadequate (or unsatisfactory)<br>5. Suboptimal (or poor)<br>95. Not applicable<br>99. None of the above or unable to determine	1,2,3,4,5,95,99<br>Will be auto-filled as 95 if nqualprep = 2<br>Cannot enter 99 with any other number<br>Cannot enter 1 or 2 with 3, 4, or 5	Select the value(s) that indicate the quality of bowel preparation documented in the procedure report/note. If the term used to describe the quality of bowel preparation is not listed or is not similar to one of the terms listed, select "99".<br>If bowel prep quality documentation is conflicting (e.g. adequate except cecum was poor), select "99".
48	nbbpsprep	Was the Boston Bowel Preparation Scale (BBPS) used to document the quality of bowel preparation?<br>3. Yes<br>No	1,2<br>If 2, go to nonbiop	**Only Acceptable Source: Colonoscopy Report or Colonoscopy Procedure Note**<br>The BBPS requires a score be assigned to each segment (right (cecum and ascending), transverse, left (descending, sigmoid and rectum)) of the colon. The segment scores range from 0 – 3 and are summed for a total BBPS score ranging from 0 -9. Segment points are assigned by the endoscopist as follows:<br>0 = unprepared colon segment with mucosa note seen due to solid stool that cannot be cleared<br>1 = Portion of mucosa of the colon segment seen, but other areas of the colon segment not well seen due to staining, residual stool and/or opaque liquid.<br>2 = Minor amount of residual staining, small fragments of stool and/or opaque liquid, but mucosa of colon segment seen well.<br>3 = Entire mucosa of colon segment seen well with no residual staining, small fragments of stool or opaque liquid.<br>**If there is documentation the BBPS was used to document the quality of bowel preparation, select "1".**

Appendix 2. Abstraction instrument for manual record review

VHA EPRP
COLORECTAL CANCER SCREENING COLONOSCOPY OVERUSE STUDY_v3
FY2019

#	Name	QUESTION	Field Format	DEFINITIONS/DECISION RULES
49	nbbpsr<br>nbbpst<br>nbbpsd	Enter the BBPS score for each colon segment documented in the colonoscopy report/procedure note.<br>**Segment** / **Score** Whole numbers 0 – 3 Abstractor may enter zz<br>1. Right colon (ascending) / ___<br>2. Transverse colon / ___<br>3. Left colon (descending) / ___		**Only Acceptable Source: Colonoscopy Report or Colonoscopy Procedure Note**<br>**Enter the BBPS score for each colon segment documented in the colonoscopy report/procedure note.**<br>If the score for a colon segment is not documented enter zz.
50	nbbpstot	Enter the total BBPS score documented in the colonoscopy report/procedure note.	Whole numbers<br>0 -9<br>May enter zz	**Only Acceptable Source: Colonoscopy Report or Colonoscopy Procedure Note**<br>Enter the BBPS total score documented in the colonoscopy report/procedure note.<br>If the total BBPS score is not documented in the colonoscopy report/procedure note, enter zz.
51	nonbiop	Was a biopsy or other tissue removal (e.g., polypectomy) performed during the non-index colonoscopy?<br>1. Yes<br>2. No	1,2<br>If 2, auto-fill nbiop as 95,and go to nordind	Review the colonoscopy procedure note/report to determine if a biopsy or polyp was removed during the procedure.<br>Suggested data source: Colonoscopy report, Colonoscopy procedure note

#	Name	QUESTION	Field Format	DEFINITIONS/DECISION RULES
52	nbiop1<br>nbiop2<br>nbiop3<br>nbiop95<br>nbiop99	Do pathology results from biopsy/tissue removal show any of the following?<br>**Indicate all that apply:**<br>1. Adenoma or adenomatous polyp of the colon or rectum<br>2. Cancer, carcinoma, or adenocarcinoma of the colon or rectum<br>3. Inflammatory bowel disease (IBD, ulcerative colitis, or Crohn's disease)<br>95. Not applicable<br>99. None of the above	1,2,3,95,99<br>Will be auto-filled as 95 if nonbiop = 2	Suggested data source: Pathology report
53	nordind	Is there physician/APN/PA documentation in the record of a specific reason (indication) the non-index colonoscopy was ordered?<br>1. Yes<br>2. No	1,2<br>If 2, auto-fill crcscrn4 as 95, nordrsk as 95, nordiag as 95, and go to nonindt2 as applicable	If the reason or indication for the non-index colonoscopy was not documented by the physician performing the procedure, look for documentation of the reason the colonoscopy was ordered in the progress notes (or consult request) of the physician/APN/PA that referred the patient for the colonoscopy.<br>Reasons (indication) for performing a colonoscopy may include, but are not limited to:<br>● Family history of colorectal cancer<br>● Family history of colorectal adenomas or polyps<br>● Personal history of polyps Personal history of colorectal cancer<br>● Inflammatory bowel disease, Crohn's disease, or ulcerative colitis<br>● High risk, NOS<br>● Surveillance, NOS<br>● Symptoms such as bleeding, change in bowel habits, abdominal pain<br>● Screening only

VHA EPRP
COLORECTAL CANCER SCREENING COLONOSCOPY OVERUSE STUDY_v3
FY2019

#	Name	QUESTION	Field Format	DEFINITIONS/DECISION RULES
54	crcscrn4	Is there physician/APN/PA documentation the non-index colonoscopy was ordered for average-risk (routine) screening only?<br>1. Yes<br>2. No<br>95. Not applicable	1,2,95<br>Will be auto-filled as 95 if nordind = 2<br>If 1, go to nonindt2 as applicable	In order to answer "1", the physician/APN/PA that referred the patient for the colonoscopy must clearly indicate the colonoscopy was ordered for average-risk screening only. It is not necessary to see the term "average-risk".<br>If documentation indicates the colonoscopy was ordered for routine screening, select "1".<br>If documentation indicates the colonoscopy was ordered due to symptoms such as bleeding, change in bowel habits, abdominal pain or any of the high-risk screening/surveillance indications listed below, enter "2".<br>High Risk Screening/Surveillance Indications:<br>● Family history of colorectal cancer<br>● Family history of colorectal adenomas or polyps<br>● Personal history of polyps Personal history of colorectal cancer<br>● Inflammatory bowel disease, Crohn's disease, or ulcerative colitis<br>● High risk, NOS<br>● Surveillance, NOS

#	Name	QUESTION	Field Format	DEFINITIONS/DECISION RULES
55	nordrsk1<br>nordrsk2<br>nordrsk3<br>nordrsk4<br>nordrsk5<br>nordrsk6<br>nordrsk7<br>nordrsk95<br>nordrsk99	Select all high-risk screening or surveillance indications documented by the ordering physician/APN/PA.<br>Indicate all that apply:<br>1. Family history of colorectal cancer<br>2. Family history of colorectal adenomas or polyps<br>3. Personal history of polyps<br>4. Personal history of colorectal cancer<br>5. Inflammatory bowel disease, Crohn's disease, or ulcerative colitis<br>6. High risk, NOS<br>7. Surveillance, NOS<br>95. Not applicable<br>99. None of the above	1,2,3,4,5,6,7,95,99<br>Will be auto-filled as 95 if nordind = 2	High-risk screening or surveillance involves testing patients that previously had colorectal cancer or are at increased risk for colorectal cancer.
56	nordiag	Was the non-index colonoscopy ordered for a non-screening diagnostic indication (bleeding, anemia, positive FOBT, diarrhea, constipation, abdominal pain, etc.)?<br>1. Yes<br>2. No<br>95. Not applicable	1,2,95<br>Will be auto-filled as 95 if nordind = 2<br>Hard edit: If nordrsk99 = -1 cannot = 2	Non-screening diagnostic indication = person has symptoms of a condition or disease<br>If any non-screening diagnostic indication is documented by the physician/APN/PA as the reason for ordering the colonoscopy, answer "1".

#	Name	QUESTION	Field Format	DEFINITIONS/DECISION RULES
		**Other Non-Index Colonoscopy**		
57	nonindt2	Enter the date of the most recent non-index colonoscopy performed prior to the most recent non-index colonoscopy.	mm/dd/yyyy<br>**Computer will pre-fill date of non-index colonoscopy prior to most recent non-index colonoscopy<br>If null and nbiop = 99, go to othpath1; else if null, go to colfobt**<br><= 10 years prior to indexdt and < nonindt1	Computer will pre-fill the date of the most recent non-index colonoscopy performed within 10 years prior to the most recent non-index colonoscopy.
58	nonindx2	On (computer to display nonindt2), is there documentation that the non-index colonoscopy was completed?<br>1. Yes<br>2. No	1,2<br>If 1, auto-fill othnon2 as 95, actnondt2 as 99/99/9999, and go to pcolnva5	Search procedure reports, surgical reports, and progress notes for documentation that a non-index colonoscopy was performed on date entered in NONINDT2.
59	othnon2	During the timeframe from (computer to display nonindt2 – 180 days to nonindt2 + 180 days), is there documentation a non-index colonoscopy was performed?<br>1. Yes<br>2. No<br>95. Not applicable	1,2,95<br>Will be auto-filled as 95 if nonindx2 = 1<br>***If 2 and nbiop = 99, go to othpath1; else, go to colfobt**	Search procedure reports, surgical reports, and progress notes for documentation that a non-index colonoscopy was performed within 180 days prior to or after the pre-filled non-index colonoscopy date.
60	actnondt2	Enter the date of the non-index colonoscopy performed during the (computer to display computer to display nonindt2 – 180 days to nonindt2 + 180 days).	mm/dd/yyyy<br>Will be auto-filled as 99/99/9999 if nonindx2 = 1<br><= 180 days prior to or after nonindt2	If more than one non-index colonoscopy was performed within 180 days prior to or after the non-index colonoscopy date, enter the date of the other colonoscopy performed closest to the date of the non-index colonoscopy.

#	Name	QUESTION	Field Format	DEFINITIONS/DECISION RULES
61	pcolnva5	Was the non-index colonoscopy performed at a VAMC?<br>1. Yes<br>2. No	1,*2<br>*If 2 and nbiop = 99, go to othpath1; else if 2, go to colfobt	If the non-index colonoscopy was performed at a VAMC, enter "1".<br>If the non-index colonoscopy was performed outside the VHA, enter "2".
62	colnrpt3	Was the non-index colonoscopy procedure report/note found in the medical record?<br>3. Procedure report found in CPRS<br>4. Procedure report found in VISTA imaging<br>99. Procedure report not found in medical record	3,4,99<br>**Warning if 99 and pcolnva5 = 1**<br>**If 99 and nbiop = 99, go to othpath1, else if 99, go to colfobt**	If the actual non-index colonoscopy procedure report/note is not found in CPRS or VISTA imaging, enter "99".<br>Suggested data sources: Notes, consults, procedures, VISTA imaging
63	anescoln3	Is there documentation that an anesthesiologist or Certified Registered Nurse Anesthetist (CRNA) was present at the time of the non-index colonoscopy to sedate the patient for the procedure?<br>1. Yes<br>2. No	1,2	**The intent of this question is to determine if the patient was sedated for the procedure under the supervision of an anesthesiologist or Certified Registered Nurse Anesthetist (CRNA).** This type of procedural sedation may be documented as Monitored Anesthesia Care (MAC).<br>Look at the colonoscopy report and any peri-procedural notes (including sedation records) available in the medical record for documentation of procedural sedation.<br>Note: may see the terms "anesthesia," "anesthesiologist," "CRNA (Certified Registered Nurse Anesthetist)", "Propofol" (under medications), "See anesthesia note," or "MAC (monitored anesthesia care)."<br>If any of the above terms are documented, select "Yes."<br>If none of these terms are present, select "No."

Appendix 2. Abstraction instrument for manual record review

#	Name	QUESTION	Field Format	DEFINITIONS/DECISION RULES
64	nonind2	Is there documentation of a specific reason (indication) the non-index colonoscopy was performed?<br>1. Yes<br>2. No	1,2<br>If 2, auto-fill crcscrn5 as 95, sothrsk as 95, and go to nordind2	**Only Acceptable Source: Colonoscopy Report or Colonoscopy Procedure Note**<br>Other terms for reason (indication) may be used (e.g., pre-operative diagnosis).<br>Reasons (indication) for performing a colonoscopy may include, but are not limited to:<br>● Family history of colorectal cancer<br>● Family history of colorectal adenomas or polyps<br>● Personal history of polyps<br>● Personal history of colorectal cancer<br>● Inflammatory bowel disease, Crohn's disease, or ulcerative colitis<br>● High risk, NOS<br>● Surveillance, NOS<br>● Symptoms such as bleeding, change in bowel habits, abdominal pain<br>● Screening only

Appendix 2. Abstraction instrument for manual record review

#	Name	QUESTION	Field Format	DEFINITIONS/DECISION RULES
65	crcscrn5	Is there physician/APN/PA documentation the non-index colonoscopy was performed for average-risk (routine) screening only?<br>1. Yes<br>2. No<br>95. Not applicable	1,2,95<br>Will be auto-filled as 95 if nonind2 = 2<br>If 1, go to nordind2	**Only Acceptable Source: Colonoscopy Report or Colonoscopy Procedure Note**<br>In order to answer "1", the physician/APN/PA documentation must clearly indicate the colonoscopy was performed for average-risk screening only.<br>It is not necessary to see the term "average-risk".<br>If documentation indicates the colonoscopy was performed for routine screening, select "1".<br>If documentation indicates the colonoscopy was performed due to symptoms such as bleeding, change in bowel habits, abdominal pain or any of the high-risk screening/surveillance indications listed below, enter "2".<br>**High Risk Screening/Surveillance Indications:**<br>● Family history of colorectal cancer<br>● Family history of colorectal adenomas or polyps<br>● Personal history of polyps Personal history of colorectal cancer<br>● Inflammatory bowel disease, Crohn's disease, or ulcerative colitis<br>● High risk, NOS<br>● Surveillance, NOS

#	Name	QUESTION	Field Format	DEFINITIONS/DECISION RULES
66	sothrsk1<br>sothrsk2<br>sothrsk3<br>sothrsk4<br>sothrsk5<br>sothrsk6<br>sothrsk7<br>sothrsk95<br>sothrsk99	Select all high-risk screening or surveillance indications documented in the non-index colonoscopy report/procedure note.<br>**Indicate all that apply:**<br>1. Family history of colorectal cancer<br>2. Family history of colorectal adenomas or polyps<br>3. Personal history of polyps<br>4. Personal history of colorectal cancer<br>5. Inflammatory bowel disease, Crohn's disease, or ulcerative colitis<br>6. High risk, NOS<br>7. Surveillance, NOS<br>95. Not applicable<br>99. None of the above	1,2,3,4,5,6,7,95,99<br>Will be auto-filled as 95 if nonind2 = 2<br>Warning if 99 and nonind2 = 1	**Only Acceptable Source: Colonoscopy Report or Colonoscopy Procedure Note**<br>Other terms for reason (indication) may be used (e.g., pre-operative diagnosis).<br>High-risk screening or surveillance involves testing patients that previously had colorectal cancer or are at increased risk for colorectal cancer.
67	nordind2	Is there physician/APN/PA documentation in the record of a specific reason (indication) the non-index colonoscopy was ordered?<br>1. Yes<br>2. No	1,2<br>If 2, auto-fill crcscrn6 as 95, sordrsk as 95, AND if nbiop = 99, go to othpath1; else, if 2, go to colfobt	If the reason or indication for the non-index colonoscopy was not documented by the physician performing the procedure, look for documentation of the reason the colonoscopy was ordered in the progress notes (or consult request) of the physician/APN/PA that referred the patient for the colonoscopy.<br>**Reasons (indication) for performing a colonoscopy may include, but are not limited to:**<br>• Family history of colorectal cancer<br>• Family history of colorectal adenomas or polyps<br>• Personal history of polyps Personal history of colorectal cancer<br>• Inflammatory bowel disease, Crohn's disease, or ulcerative colitis<br>• High risk, NOS<br>• Surveillance, NOS<br>• Symptoms such as bleeding, change in bowel habits, abdominal pain<br>• Screening only

#	Name	QUESTION	Field Format	DEFINITIONS/DECISION RULES
68	crcscrn6	Is there physician/APN/PA documentation the non-index colonoscopy was ordered for average-risk (routine) screening only?<br>1. Yes<br>2. No<br>95. Not applicable	1,2,95<br>Will be auto-filled as 95 if nordind2 = 2<br>If 1, go to nonbiop2	In order to answer "1", the physician/APN/PA that referred the patient for colonoscopy must clearly indicate the colonoscopy was ordered for average-risk screening only.<br>It is not necessary to see the term "average-risk".<br>If documentation indicates the colonoscopy was ordered for routine screening, select "1".<br>If documentation indicates the colonoscopy was ordered due to symptoms such as bleeding, change in bowel habits, abdominal pain or any of the high-risk screening/surveillance indications listed below, enter "2".<br>**High Risk Screening/Surveillance Indications:**<br>● Family history of colorectal cancer<br>● Family history of colorectal adenomas or polyps<br>● Personal history of polyps<br>● Personal history of colorectal cancer<br>● Inflammatory bowel disease, Crohn's disease, or ulcerative colitis<br>● High risk, NOS<br>● Surveillance, NOS

Appendix 2. Abstraction instrument for manual record review

#	Name	QUESTION	Field Format	DEFINITIONS/DECISION RULES
69	sordrsk1<br>sordrsk2<br>sordrsk3<br>sordrsk4<br>sordrsk5<br>sordrsk6<br>sordrsk7<br>sordrsk95<br>sordrsk99	Select all high-risk screening or surveillance indications documented by the ordering physician/APN/PA.<br>**Indicate all that apply:**<br>1. Family history of colorectal cancer<br>2. Family history of colorectal adenomas or polyps<br>3. Personal history of polyps<br>4. Personal history of colorectal cancer<br>5. Inflammatory bowel disease, Crohn's disease, or ulcerative colitis<br>6. High risk, NOS<br>7. Surveillance, NOS<br>95. Not applicable<br>99. None of the above	1,2,3,4,5,6,7,95,99<br>Will be auto-filled as 95 if nordind2 = 2	High-risk screening or surveillance involves testing patients that previously had colorectal cancer or are at increased risk for colorectal cancer.
70	nonbiop2	Was a biopsy or other tissue removal (e.g., polypectomy) performed during the non-index colonoscopy?<br>1. Yes<br>2. No	1,2<br>If 2, auto-fill sbiop as 95 and go to othpath1	Review the colonoscopy procedure note/report to determine if a biopsy or polyp was removed during the procedure.<br>Suggested data source: Colonoscopy report, Colonoscopy procedure note
71	sbiop1<br>sbiop2<br>sbiop3<br>sbiop95<br>sbiop99	Do pathology results from biopsy/tissue removal show any of the following?<br>**Indicate all that apply:**<br>**1.** Adenoma or adenomatous polyp of the colon or rectum<br>**2.** Cancer, carcinoma, or adenocarcinoma of the colon or rectum<br>**3.** Inflammatory bowel disease (IBD, ulcerative colitis, or Crohn's disease)<br>95. Not applicable<br>99. None of the above	1,2,3,95,99<br>Will be auto-filled as 95 if nonbiop2 = 2<br>If 1,2,or 3, auto-fill othpath as 95, and go to colfobt	Suggested data source: Pathology report

Appendix 2. Abstraction instrument for manual record review

#	Name	QUESTION	Field Format	DEFINITIONS/DECISION RULES
72	othpath1<br>othpath2<br>othpath3<br>othpath95<br>othpath99	During the timeframe from (computer to display indexdt - 10 years to indexdt – 1 day), does VHA anatomic/surgical pathology data document a history of any of the following high-risk features?<br>**Indicate all that apply:**<br>**1.** Adenoma or adenomatous polyp of the colon or rectum<br>**2.** Cancer, carcinoma, or adenocarcinoma of the colon or rectum<br>**3.**Inflammatory bowel disease (IBD, ulcerative colitis, or Crohn's disease)<br>95. Not applicable<br>99. None of the above	1,2,3,95,99<br>Will be auto-filled as 95 if sbiop = 1,2, or 3<br>If 99, auto-fill othpathdt as 99/99/9999	Search anatomic/surgical pathology data for any reports on colon/rectal specimens during the 10 years prior to the index colonoscopy.<br>**DO NOT INCLUDE PATHOLOGY RESULTS FROM THE INDEX COLONOSCOPY.**
73	othpathdt	Enter the date of the earliest report documenting high-risk colon/rectal pathology features.	mm/dd/yyyy<br>Will be auto-filled as 99/99/9999 if othpath = 99<br><= 10 years prior to indexdt and < indexdt	If more than one anatomic/surgical pathology report documents high-risk colon/rectal pathology features, enter the date of the earliest report.<br>Enter the exact date.
		**Fecal Occult Blood Testing**		
74	colfobt	Does the medical record contain the result(s) of any fecal occult blood testing done during the timeframe from (computer to display indexdt – 12 months to indexdt – 1 day)?<br>3. FOBT done in VHA<br>4. FOBT done in private sector<br>99**.** No result of FOBT done within the past 12 months	3,4,*99<br>***If 99, go to end**	The FOBT result(s) must be documented in the medical record during the 12 months prior to the index colonoscopy. Patient self-report of FOBT is NOT acceptable.<br>Suggested data sources: Lab reports, VISTA imaging

#	Name	QUESTION	Field Format	DEFINITIONS/DECISION RULES
75	cfobtdt	Enter the date of the most recent FOBT result(s) reported.	mm/dd/yyyy<br><= 12 months prior to indexdt and < indexdt	Enter the exact date.
76	fobtype	What type of FOBT was performed?<br>1. Guaiac (gFOBT)<br>2. Immunochemical (iFOBT/FIT)<br>99. Unable to determine	1,2,99	If unable to determine whether the fecal occult blood testing was guaiac (gFOBT) or immunochemical (iFOBT), select “99”.
77	ifobtnum	Enter the number of FOBT results reported in the record on (computer to display cfobtdt).	____<br>Whole numbers 1 - 3	Enter the exact number of FOBT results reported on the date entered in CFOBTDT.
78	cfobtpos	Was at least <u>one result</u> of the most recent FOBT (or set of screening FOBTs) reported as positive?<br>1. Yes<br>2. No	1,2	If at least one FOBT test result was reported as positive, select “1”. If all reported results were negative, select “2”.
